# Changes in bumblebee queen gut microbiotas during and after overwintering diapause

**DOI:** 10.1111/imb.12957

**Published:** 2024-08-22

**Authors:** Michelle Z. Hotchkiss, Jessica R. K. Forrest, Alexandre J. Poulain

**Affiliations:** ^1^ Department of Biology University of Ottawa Ottawa Ontario Canada

**Keywords:** *Bombus*, bumblebees, diapause, gut microbiotas, metagenomics, overwintering, pesticide exposure, social bee gut microbiota

## Abstract

Bumblebees are key pollinators with gut microbiotas that support host health. After bumblebee queens undergo winter diapause, which occurs before spring colony establishment, their gut microbiotas are disturbed, but little is known about community dynamics during diapause itself. Queen gut microbiotas also help seed worker microbiotas, so it is important that they recover post‐diapause to a typical community structure, a process that may be impeded by pesticide exposure. We examined how bumblebee queen gut microbiota community structure and metabolic potential shift during and after winter diapause, and whether post‐diapause recovery is affected by pesticide exposure. To do so, we placed commercial *Bombus impatiens* queens into diapause, euthanizing them at 0, 2 and 4 months of diapause. Additionally, we allowed some queens to recover from diapause for 1 week before euthanasia, exposing half to the common herbicide glyphosate. Using whole‐community, shotgun metagenomic sequencing, we found that core bee gut phylotypes dominated queen gut microbiotas before, during and after diapause, but that two phylotypes, *Schmidhempelia* and *Snodgrassella*, ceased to be detected during late diapause and recovery. Despite fluctuations in taxonomic community structure, metabolic potential remained constant through diapause and recovery. Also, glyphosate exposure did not affect post‐diapause microbiota recovery. However, metagenomic assembly quality and our ability to detect microbial taxa and metabolic pathways declined alongside microbial abundance, which was substantially reduced during diapause. Our study offers new insights into how bumblebee queen gut microbiotas change taxonomically and functionally during a key life stage and provides guidance for future microbiota studies in diapausing bumblebees.

## Graphical abstract

- We used shotgun metagenomic sequencing to examine how bumblebee (*B. impatiens*) queen gut microbiota community structure shifted during and after overwintering diapause.
- Microbial abundance in queen microbiotas declined rapidly during a 4‐month diapause and did not fully recover by the end of a 1‐week post‐diapause recovery period.
- Core social bee gut microbial phylotypes dominated queen gut microbiotas before, during and after diapause, but two phylotypes, *Schmidhempelia* and *Snodgrassella*, ceased to be detected during late diapause and recovery.

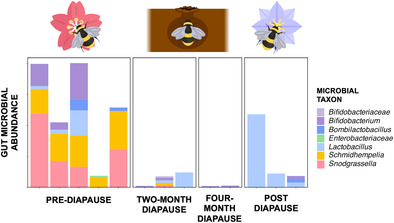

## INTRODUCTION

Bumblebees are key pollinators in wild and agricultural ecosystems. Their health, like that of other eusocial, corbiculate bees, is supported by a core microbial community living in their hindgut (Hammer, Le, Martin, & Moran, 2021; Kwong & Moran, 2016; Motta & Moran, 2024). The social bee gut microbiota contains five core phylotypes: *Bifidobacterium* spp., *Bombilactobacillus* spp., *Gilliamella* spp., *Lactobacillus* spp. near *melliventris* and *Snodgrassella* spp.; the first four phylotypes primarily ferment sugars provided by the host diet and the last metabolizes sugar fermentation products (Kwong & Moran, 2016). These phylotypes are well conserved both within and across eusocial corbiculate bee tribes (i.e., honeybees, stingless bees and bumblebees). However, certain host lineages have gained additional core phylotypes—such as *Candidatus* Schmidhempelia spp. (hereafter *Schmidhempelia*) in many bumblebee species—or lost others (Cerqueira et al., 2021; Kwong, Medina, et al., 2017) and phylotypes can also show host specificity at the strain and species levels (Kwong et al., 2014; Powell et al., 2016). The social bee gut microbiota primarily benefits its hosts through immune system stimulation (Horak et al., 2020; Kwong, Mancenido, & Moran, 2017; Lang et al., 2022; Steele et al., 2017) and defence against pathogens (Cariveau et al., 2014; Koch & Schmid‐Hempel, 2011; Miller et al., 2021; Palmer‐Young et al., 2019), though it can also synthesize hormones (Kešnerová et al., 2017), process nutrients (Engel et al., 2012; Kešnerová et al., 2017; Lee et al., 2018; Zheng et al., 2017, 2019) and aid in detoxification (Rothman et al., 2019; Wu et al., 2020). Given these benefits, it is important for social bees to maintain their gut microbiotas. However, bumblebee ecology presents an inherent obstacle to this maintenance in the form of winter diapause.

The three tribes of eusocial corbiculate bees differ in how they withstand periods of adverse environmental conditions, and, consequently, how their gut microbiotas shift during these periods. Both honeybees and stingless bees form perennial colonies in which bees consume food (i.e., nectar, pollen and/or honey) year‐round (Dos Santos et al., 2014), continuously feeding both themselves and their gut microbes. Additionally, both tribes consistently maintain warm colony temperatures (Jones & Oldroyd, 2006), even through winter in temperate honeybee colonies (Heinrich, 1981). While seasonal changes in stingless bee gut microbiotas have yet to be examined, winter honeybee gut microbiotas generally contain the same core phylotypes as summer microbiotas, though at different relative and absolute abundances, and total microbial abundance is the same as or higher than in summer microbiotas (Castelli et al., 2022; Kešnerová et al., 2020; Li et al., 2022).

In contrast to honeybees and stingless bees, bumblebee species in temperate environments have annual colony life cycles. In late summer, colonies produce a new generation of queens. Newly eclosed queens remain a few days in their natal colonies, acquiring gut microbes from their nest‐mates and nest environment (Billiet et al., 2017; Hammer, Le, Martin, & Moran, 2021), before leaving to mate. After mating, they forage on pollen and nectar to accumulate lipid and glycogen stores, then dig hibernacula and enter winter diapause (Carnell et al., 2020; Röseler & Röseler, 1986; Woodard et al., 2019). During this period, which can last 4–10 months, depending on the bumble bee species (Heinrich, 2004), queen body temperatures fall to a few degrees Celsius (Alford, 1969; Heinrich, 2004; Keaveny et al., 2022; Yoon et al., 2010, 2013) and they consume no food. Bumblebee core gut microbes spend non‐diapause months living in a nutrient‐rich gut environment at temperatures at least a few degrees higher than ambient air temperature (Heinrich, 1977), so diapause conditions present a challenge that is unique among social bee tribes. Thus, unsurprisingly, bumblebee gut microbiotas experience seasonal shifts in community structure (Bosmans, Pozo, Verreth, Crauwels, Wäckers, et al., 2018).

While we know that diapause alters bumblebee gut microbiotas, overall, little is known about community fluctuations during this period. One study found high relative abundances of core gut microbes in *Bombus terrestris* guts after diapause, but lacked profiles of pre‐diapause communities for comparison (Su et al., 2021). Meanwhile, a study in *B. impatiens* found that diversity and relative abundance of non‐core microbes increased after diapause while total microbial abundance declined by approximately 50% (Bosmans, Pozo, Verreth, Crauwels, Wäckers, et al., 2018), but only ileal (i.e., upper hindgut) microbiotas were examined, and only immediately before and after diapause. At least in honey bees, three core social bee phylotypes (*Bifidobacterium* spp., *Bombilactobacillus* spp. and *Lactobacillus* spp. near *melliventris*) seem to reside primarily in the rectum, the lower bee hindgut. Therefore, to date, we have an incomplete picture of how bumblebee gut microbiota community structure changes during and after diapause, and it is also unknown whether taxonomic changes are accompanied by shifts in microbiota metabolism.

Furthermore, little is known about bumblebee gut microbiota community dynamics after diapause. When bumblebee queens emerge from diapause in spring, each finds a new colony, raising the first generation of workers on her own. As bumblebee gut microbiotas are primarily seeded through interactions with conspecifics and hive materials (Billiet et al., 2017; Hammer, Le, Martin, & Moran, 2021; Su et al., 2021), bumblebee queens are a key source of gut microbes for their workers. Post‐diapause recovery of the queen gut microbiota to a ‘standard’ community structure is thus important to the health of the colony as a whole. One study that sampled wild *B. terrestris* queen ileal microbiotas in early spring (i.e., soon after the end of diapause) found high relative abundances of the core gut microbes *Snodgrassella* and *Gilliamella* (Bosmans, Pozo, Verreth, Crauwels, Wilberts, et al., 2018). Other studies examining gut microbiotas of lab‐reared queens 1–3 months after diapause emergence found that communities are dominated by core gut microbes (Wang et al., 2019), but that some phylotypes, such as *Gilliamella*, may be lost (Koch et al., 2013). These studies imply that post‐diapause recovery of queen gut microbiotas is possible. However, none of these studies profiled gut microbiotas during diapause, nor did they investigate microbial community dynamics early in recovery. Moreover, as wild queens must forage in the early spring to feed both themselves and their offspring, they may be exposed to stressors that could impede post‐diapause gut microbiota recovery, such as pesticides (Hotchkiss et al., 2022).

In this study, we use metagenomics to compare bumblebee (*B. impatiens*) queen gut microbiota taxonomic community structure and metabolic potential before, during and after diapause to better understand bumblebee gut microbiota community dynamics during this key stage in the colony life cycle. Additionally, we compare post‐diapause bumblebee queen gut microbiota recovery in the presence and absence of an external stressor, glyphosate exposure. Glyphosate is the most used herbicide globally (Benbrook, 2016; Duke & Powles, 2008), and exposure to this compound consistently decreases the abundance of *Snodgrassella alvi* in social bee gut microbiotas (Blot et al., 2019; Cullen et al., 2023; Helander et al., 2023; Motta et al., 2018, 2020; Motta & Moran, 2020, 2023b; Straw et al., 2023). *Snodgrassella alvi* resides primarily in the ileum where it forms a biofilm, and likely cross‐feeds, with *Gilliamella* spp. (Hammer, Le, Martin, & Moran, 2021). In honey bees, *S. alvi* has been shown to help maintain an anoxic environment that supports the growth of other core, anaerobic microbes (Engel et al., 2013; Kwong et al., 2014; Zheng et al., 2017). We, therefore, expected that glyphosate exposure could interfere with post‐diapause microbiota community dynamics.

## RESULTS

To examine changes in queen bumblebee gut microbiotas during and after diapause, we placed *Bombus impatiens* queens into one of five time‐points/treatments (hereafter ‘treatments’): three different diapause durations (0‐month/pre‐diapause, 2‐month and 4‐month diapause treatments), plus two post‐diapause recovery treatments, in which queens experienced 4 months of diapause and were then fed either a sugar‐plus‐glyphosate solution (recovery + glyphosate treatment) or an unspiked sugar solution (recovery control) over a 1‐week period. At the end of each treatment, we euthanized queens and sampled their full guts. We performed shotgun metagenomic sequencing on five queen gut samples per treatment to determine gut microbiota taxonomic composition and metabolic potential. We also used quantitative polymerase chain reaction (qPCR) to determine the number of 16S rRNA gene copies, a proxy for microbial abundance, in each sequenced microbiota (*n* = 5 per treatment).

### Microbial abundance through diapause

**FIGURE 1:**
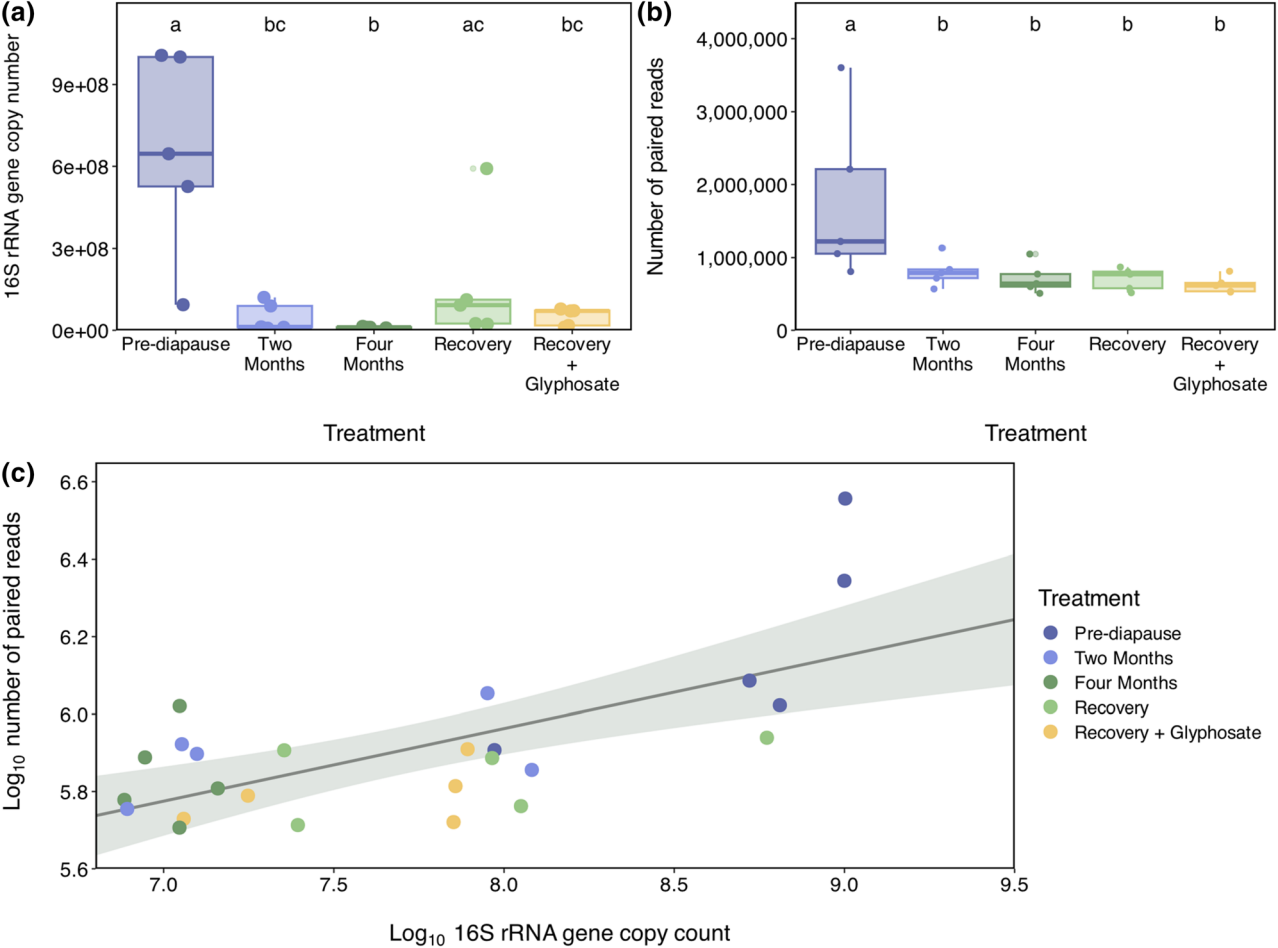
(a) Box plot of total 16S rRNA gene copy counts in queen *B. impatiens* gut microbiotas before, during and after diapause. (b) Box plot of the number of paired reads remaining after host and plant filtering in sequencing libraries of queen *B. impatiens* gut microbiotas sampled before, during and after diapause. Boxes represent medians and interquartile ranges; whiskers extend to 1.5 × the interquartile range. Treatments with different letters are significantly different based on a Tukey post‐hoc test. (c) Correlation between log10 16S rRNA gene copy counts and log10 number of paired reads remaining after host and plant filtering for queen *B. impatiens* gut microbiotas. Trendline is drawn using the lm (‘linear model’) smoothing method in ggplot2. For all panels, *n* = 25 (*n* = 5 per treatment).

We found that 16S rRNA gene copy count varied with treatment (*F*_4,18_ = 10.8; *p* < 0.001) but not among natal colonies (*F*_2,18_ = 1.74, *p* = 0.20). Specifically, pairwise comparisons showed 16S rRNA gene copy counts in pre‐diapause microbiotas were higher than those in 2‐month diapause, 4‐month diapause and recovery + glyphosate treatments (means: pre‐diapause = 6.5 × 10^8^, 2‐month diapause = 4.8 × 10^7^, 4‐month diapause = 1.1 × 10^7^, recovery + glyphosate = 5.0 × 10^7^; all *p* < 0.05; Figure 1a). Gene counts of microbiotas from the recovery control treatment were also higher than those of queen microbiotas in the 4‐month diapause treatment (means: 4‐month diapause = 1.1 × 10^7^, recovery control = 1.7 × 10^8^; *p* = 0.03; Figure 1a). However, the recovery control treatment contained an extreme data point with a gene copy count of almost 3.5× that of the treatment mean. To determine the robustness of our results, we examined pairwise comparisons again with this data point removed. While treatment still affected gene copy count (*F*_4,17_ = 13.5, *p* < 0.001), pre‐diapause gene copy counts were now higher than those in all other treatments (all *p* < 0.01), and all other pairwise comparisons were insignificant.

The number of paired reads after removing host and plant sequences also differed among treatments (*F*_4,18_ = 6.56, *p* = 0.002) but not among colonies (*F*_2,18_ = 2.85, *p* = 0.08), with the number of remaining paired reads higher in pre‐diapause queens than in any other treatment (all *p* < 0.05; Figure 1b). Gene copy counts and the number of paired reads post‐filtering were correlated (*r* = 0.67, *p* < 0.001; Figure 1c).

### Taxonomic profiles through diapause

**FIGURE 2:**
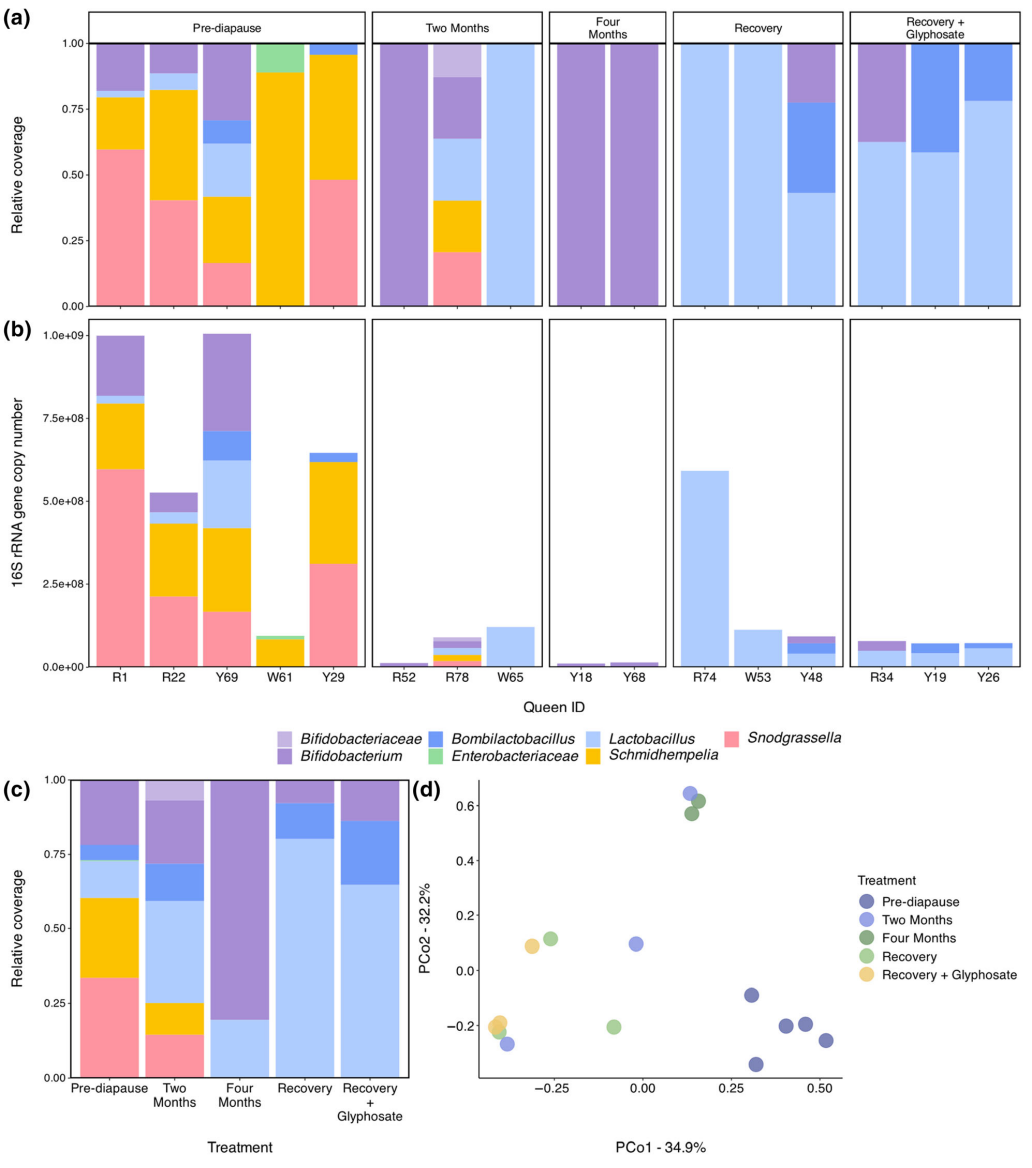
Taxonomic community structure of bumble bee (*B. impatiens*) queen gut microbiotas before, during and after diapause. (a) Stacked bar plot of relative abundances of microbial phylotypes in microbiotas faceted by treatment (*n* = 16; *n* = 2–5 per treatment). Queen IDs consist of a letter corresponding to the queen's natal colony and a number. (b) Stacked bar plot of 16S rRNA gene copy counts of microbial phylotypes in bumble bee queen gut microbiotas faceted by treatment (*n* = 16; *n* = 2–5 per treatment). (c) Stacked bar plot of relative abundances of microbial phylotypes in bumble bee queen gut microbiota coassemblies (coassembled by treatment) (*n* = 5; *n* = 1 per treatment). (d) Principal coordinates analysis of bumble bee queen gut microbiotas (*n* = 16; *n* = 2–5 per treatment) using Bray–Curtis dissimilarities. For all panels, taxonomy was assigned in anvio using ribosomal protein S7. Community structure varies with treatment (PERMANOVA: *F*_4,9_ = 4.4, *p* = 0.0003) but not natal colony ID (PERMANOVA: *F*_2,9_ = 1.8, *p* = 0.09); pre‐diapause gut microbiotas had significantly different community structure than all other treatments (all *F* > 3.5, all *p* < 0.05).

We assigned taxonomy to metagenomic assemblies using the single‐copy gene ribosomal protein S7. Of our 25 individual assemblies with bacterial contigs, we did not find ribosomal protein S7 sequences in nine. All assemblies without S7 sequences belonged to gut microbiotas sampled during or after diapause with <3.0 × 10^8^ 16S rRNA gene copies, and all but one of these assemblies contained fewer than 100 bacterial contigs. Across the 16 microbiotas where taxonomy could be assigned (*n* = 2–5 per treatment), we identified seven taxa: six were core bumblebee gut phylotypes—*Bifidobacterium* (family *Bifidobacteriaceae*), *Bombilactobacillus*, *Lactobacillus*, *Schmidhempelia* and *Snodgrassella*—and the last was the non‐core phylotype *Enterobacteriaceae* (Figure 2a,b). In all but one profiled gut microbiota, the only taxa identified were core phylotypes. The same taxa were also observed in the coassemblies by treatment (Figure 2c), though one phylotype, *Lactobacillus*, that was absent in all individual 4‐month assemblies was identified in the 4‐month coassembly. We did not find the core phylotype *Gilliamella* in any of our queen gut microbiotas, though its absence has been noted in commercial *B. impatiens* colonies in other studies (Hotchkiss et al., 2024; Motta & Moran, 2023). Alpha diversity did not vary with treatment (Figure S5), whether the dependent variable was ranked or unranked (all *F*_4,9_ < 2.5, all *p* > 0.10), nor did it vary with natal colony ID (all *F*_2,9_ < 2.6, all *p* > 0.10). Taxonomic profiles assigned with ribosomal protein S2 were similar to those assigned with S7, though relative coverages differed slightly and some individual assemblies lost or gained a phylotype (Figures 2a and S6A). Coassembly profiles were also similar across both single copy genes, though *Lactobacillus* was identified at 4 months using protein S7 but not S2 (Figures 2c and S6B).

Microbial community structure varied with treatment (permutational analyses of variance [PERMANOVA]: *F*_4,9_ = 4.4, *p* = 0.0003; homogeneity of dispersions: *F*_4,11_ = 2.37, *p* = 0.12) but not among natal colonies (PERMANOVA: *F*_2,9_ = 1.8, *p* = 0.09; Figure 2d); the same result was observed with the taxonomic profiles assigned using ribosomal protein S2 (treatment: *F*_4,8_ = 3.5, *p* = 0.0001; natal colony ID: *F*_2,8_ = 1.9, *p* = 0.067; homogeneity of dispersions: *F*_4,10_ = 0.94, *p* = 0.47; Figure S6C). Specifically, the community structure of microbiotas before diapause was significantly different from that of any other treatment (all *F*_1,4_ > 3.5, all *p* < 0.05). The two recovery treatments did not differ in community structure (*F*_1,4_ = 1.12, *p* = 0.4). Four phylotypes, *Bombilactobacillus, Lactobacillus, Schmidhempelia* and *Snodgrassella*, were differentially abundant between treatments in the analysis of community structures of microbiomes with bias correction (ANCOMBC) global test (all W > 15.5, all *q* < 0.01). Correcting for multiple comparisons, we found that *Schmidhempelia* 16S rRNA gene copy counts were significantly higher in pre‐diapause microbiotas than in 4‐month diapause, recovery control, or recovery + glyphosate microbiotas (all W < −6.20, all *q* < 0.005), and that *Snodgrassella* 16S rRNA gene copy counts were significantly higher in pre‐diapause microbiotas than in 4‐month or recovery + glyphosate microbiotas (all W < −4.95, all *q* < 0.02). No pairwise comparisons for *Bombilactobacillus* or *Lactobacillus* were significant.

### Metabolic profiles through diapause

**FIGURE 3:**
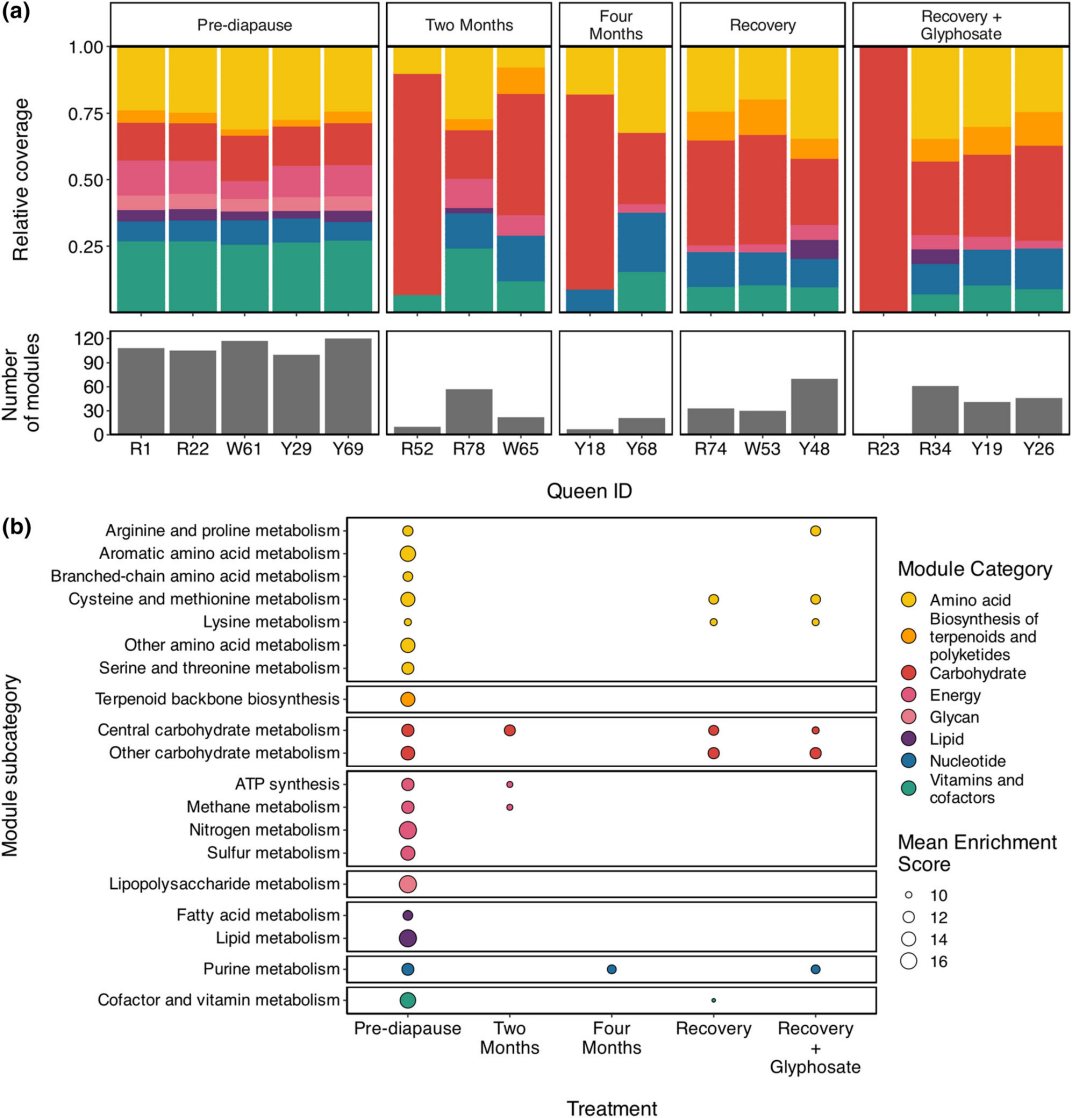
Metabolic potential of bumble bee (*B. impatiens*) queen gut microbiotas before, during and after diapause. (a) Stacked bar plot of relative coverages of KEGG module categories (i.e., metabolism types) in individual queen gut microbiotas faceted by treatment (*n* = 16; *n* = 2–5 per treatment). Beneath is a bar plot of the number of KEGG modules identified in each microbiota; for R23, number of modules = 1. Queen IDs consist of a letter corresponding to the queen's natal colony and a number. (b) Plot of significantly enriched KEGG modules in queen gut microbiota treatments, grouped by module subcategory and faceted by module category. Treatments have a point for each module that is enriched in that treatment. Point size corresponds to mean enrichment score for a given module subcategory; the higher the enrichment score, the more strongly associated the given metabolism is with the treatment.

Of the 25 queen gut microbiotas we examined, metabolic modules with ≥50% pathwise completeness could not be found in eight, and one microbiota only contained one pathway; these nine microbiotas were the same nine, which contained no copies of the ribosomal protein S7 or S2 genes in our taxonomic analyses. In the 16 remaining microbiotas (*n* = 2–5 per treatment), we identified 143 modules with ≥50% pathwise completeness from eight Kyoto Encyclopedia of Genes and Genomes (KEGG) module categories (Figure 3a). The number of modules identified per microbiota ranged from 1 to 120, and pre‐diapause microbiotas had the highest module counts (Figure 3a). At the level of KEGG module category, microbiotas did not differ in metabolic structure by treatment (PERMANOVA: *F*_4,10_ = 1.8, *p* = 0.14; homogeneity of dispersions: *F*_4,12_ = 1.06, *p* = 0.43) or natal colony (PERMANOVA: *F*_2,10_ = 0.26, *p* = 0.92). Metabolic profiles were similar for coassemblies by treatment (Figure S7).

We found 63 KEGG modules from 19 module subcategories that were differentially enriched across treatments (Figures 3b and S8). All differentially enriched modules were enriched in the pre‐diapause treatment; of those, 11 were also enriched in the recovery treatment, 10 in the recovery + glyphosate treatment, four in the 2‐month diapause treatment and one in the 4‐month diapause treatment (Figure S8).

## DISCUSSION

### Microbial abundance decreases through diapause

We found that average microbial abundance in bumblebee queen gut microbiotas decreases during diapause by an order of magnitude, falling from 6.5 × 10^8^ 16S rRNA gene copies before diapause to approximately 1.7 × 10^7^ after 4 months (Figure 1a). Additionally, we found little difference in average gene copy counts between 2 and 4 months into diapause, implying that microbial abundance declines within the first 2 months of diapause and then plateaus. Studies on gut microbiotas in analogous animal systems, like fasting hamsters and hibernating ground squirrels, observe declines in abundance at magnitudes comparable to or larger than ours (Sonoyama et al., 2009; Stevenson et al., 2014), though a study in *B. terrestris* queens observed a smaller decline (Bosmans, Pozo, Verreth, Crauwels, Wäckers, et al., 2018). While this discrepancy could be due to the difference in study species, it may also be due to differences in mating status. Bosmans et al. used mated queens in their study, while our queens were unmated. In bumblebee queens, pre‐diapause mating increases antimicrobial peptide (AMP) production, which remains elevated through diapause (Barribeau & Schmid‐Hempel, 2017; Colgan et al., 2019). AMPs play an important role in regulating gut microbiotas (Ostaff et al., 2013), and therefore whether production is elevated (i.e., whether queens are mated or unmated) could influence gut microbiota abundance and community dynamics through diapause.

We believe two factors are primarily driving gut microbial abundance decline during diapause. The first is a decline in nutrients that fuel microbial growth, such as carbohydrates from nectar and pollen and host organic acids (Hammer, Le, Martin, & Moran, 2021; Kwong & Moran, 2016; Martinson et al., 2014; Motta & Moran, 2024; Quinn et al., 2024). The second is a decline in temperature as bumblebee body temperature falls below optimal growth temperatures and lower thermal limits of core microbes (Engel et al., 2013; Hammer, Le, & Moran, 2021; Hammes & Hertel, 2009; Palmer‐Young et al., 2023; Shah, 2007). Together, these factors likely impede the growth of many bumblebee gut microbes during diapause.

After diapause, we observe an approximately eight‐fold increase in average microbial abundance over the course of 1 week, though abundance remains more than six times lower than before diapause (Figure 1a). However, microbial abundance within recovery control queens varies substantially (range: 2.3 × 10^7^–5.2 × 10^8^), as seen in other animal microbiomes during post‐disturbance recovery (Ng et al., 2019; Stevenson et al., 2014). Still, there is evidence from other studies that bumblebee queen gut microbial populations eventually return to pre‐diapause abundance. Studies of wild and lab‐reared queens found 16S rRNA gene copy counts comparable to our pre‐diapause counts in queen gut microbiotas within a few weeks after diapause (Bosmans, Pozo, Verreth, Crauwels, Wilberts, et al., 2018; Wang et al., 2019). An interesting avenue for future studies would be to examine gut microbiota recovery at a fine timescale for longer, and with larger sample sizes, than we did here, to attempt to capture the full regrowth of microbial populations after diapause.

The low microbial abundances in queen gut microbiotas during and after diapause caused difficulties during sequencing and data analysis. As we extracted DNA from whole‐gut samples without first enriching for prokaryotic cells, the ratio of host and plant DNA to microbiota DNA was higher in diapause and recovery samples than pre‐diapause samples. Consequently, even after removing reads that mapped to host and plant genomes, most contigs in our metagenomic assemblies are taxonomically assigned to invertebrates and plants (Figure S1 and Tables S2–S5). In host‐microbiome systems, when samples contain a high proportion of host DNA and abundances of target microbial taxa are low, metagenomic sequencing can fail to detect target taxa (Pereira‐Marques et al., 2019). Based on this information and our own data validations (see below), it is likely that many queen gut microbiotas in our study are not fully taxonomically or metabolically profiled. Our discussion takes this caveat into account, but there remain three key findings that we believe stand despite this limitation: (1) taxonomic community structure shifts during diapause and does not recover after 1 week, (2) metabolic potential remains constant through diapause stages and (3) glyphosate does not affect early post‐diapause gut microbiota recovery.

### Taxonomic community structure shifts during diapause

Taxonomic community structure in queen bumblebee gut microbiotas shifts after diapause onset and does not return to a pre‐diapause structure after 1 week of recovery (Figure 2). Before diapause, queen microbiotas are dominated by five core phylotypes: *Bifidobacterium* spp., *Bombilactobacillus* spp., *Lactobacillus* spp., *Schmidhempelia* spp. and *Snodgrassella* spp. After diapause begins, core phylotypes remain dominant, though some are lost in certain individual microbiotas (Figure 2). Lost phylotypes are likely not extinct, but instead have abundances below our detection limit. Generally, in our data, the fewer microbes present in gut microbiota, the fewer bacterial sequences present in a sequencing library and, consequently, the lower the assembly quality and ability to detect bacterial taxa (Tables S1, S4 and S5). Indeed, we fail to taxonomically profile any bacteria in nine samples, despite them containing positive, albeit low, 16S rRNA gene counts (Figure 1a). Additionally, we detect taxa in the coassemblies of low‐abundance treatments that are absent in all constituent individual assemblies (Figures 2c and S6B). Together, these observations, alongside our small sample sizes, indicate that the patterns we observe in taxonomic structure across treatments could be an artefact of inadequate microbial sequencing.

Still, we believe that the taxa we detect in our queen microbiotas are the dominant constituents of these communities, and that our data thus present evidence of a shift in community structure, specifically a decline in the abundance of *Schmidhempelia* and *Snodgrassella* spp. In pre‐diapause queen microbiotas, these two phylotypes consistently comprise a large proportion of the relative coverage (Figures 2a and S6A). If community structure was stable through time, we would expect *Schmidhempelia* and *Snodgrassella* to remain among the most common taxa during and after diapause, and therefore among the most detected. However, we fail to detect these phylotypes at all past 2 months of diapause, and communities are instead dominated by *Bifidobacterium, Bombilactobacillus* and *Lactobacillus*. When sequencing our microbiotas, we used PCR‐free library preparation and, excluding *Bifidobacterium* spp., GC content is similar between core phylotypes, so it is unlikely that biased sequencing explains this pattern (Browne et al., 2020) (Table S6). Likewise, bias towards Gram‐negative microbes during DNA extraction would have increased the detection of *Schmidhempelia* and *Snodgrassella* relative to other core phylotypes (Frostegård et al., 1999). For these reasons, we believe that *Schmidhempelia* and *Snodgrassella* abundance does decline during diapause and does not recover by 1 week after diapause ends. While these phylotypes are likely still present (Su et al., 2021), it is possible that they become extinct during diapause, as can occur with *Gilliamella* spp., another core bee phylotype closely related to *Schmidhempelia* (Koch et al., 2013; Martinson et al., 2014). Future studies with larger sample sizes could use more sensitive bacterial taxonomic profiling methods to investigate this possibility.

It is unclear why *Schmidhempelia* and *Snodgrassella* would be more likely to decline or die off during diapause than other phylotypes. In other vertebrate guts, *Proteobacteria* (the phylum containing *Schmidhempelia* and *Snodgrassella*) tend to increase in relative abundance during hibernation and fasting (Greene et al., 2022; Maurice et al., 2015; Sonoyama et al., 2009; Stevenson et al., 2014; Xiao et al., 2019), though this pattern is less consistent in overwintering insects (Ferguson et al., 2018; Hou et al., 2021; Wang et al., 2017) and appears taxon‐specific (Ferguson et al., 2018). In our system, the decrease in relative and absolute abundances of *Schmidhempelia* and *Snodgrassella* may be driven by differences in cold sensitivity between Gram‐negative and Gram‐positive bacteria (Schwab et al., 2014), higher lower thermal limits for growth (Hammer, Le, & Moran, 2021; Hammes & Hertel, 2009; Shah, 2007) and/or increased sensitivity to changes in available exogenous nutrients and host metabolites during diapause (Quinn et al., 2024).

Comparing our results to the only previous study that examined bumblebee queen gut microbiotas before and immediately after diapause, there are clear similarities and differences. Like us, Bosmans, Pozo, Verreth, Crauwels, Wäckers, et al. (2018) saw a decrease in core phylotype abundance after a 4‐month diapause. However, they also observed an increase in non‐core microbial abundance and diversity (Bosmans, Pozo, Verreth, Crauwels, Wäckers, et al., 2018), while we fail to detect any non‐core microbes once diapause begins (Figure 2). It is possible that microbiotas in our study contain non‐core phylotypes, but that abundances are too low for our method to detect; indeed, abundances of individual non‐core taxa were low in the Bosmans et al. study, even if, collectively, these taxa comprised large proportions of microbiotas. It should also be noted that both we and Bosmans, Pozo, Verreth, Crauwels, Wäckers, et al. (2018) reared and incubated queens under sterile conditions, and so the non‐core (i.e., environmental) microbial communities in these studies likely do not reflect those of wild bumblebee queens. Investigations of taxonomic shifts in queen gut microbiotas during diapause in more field‐realistic conditions, such as non‐sterile soil and leaf litter, should be a priority.

By 1 week after diapause, we find little evidence of community structure recovery in queen gut microbiotas (Figure 2). However, we believe that the taxonomic shifts that occur during diapause in bumblebee queen microbiotas are temporary, unlike the permanent changes observed in other insect microbiotas (Ferguson et al., 2018). Studies surveying lab‐reared and wild bumblebee queens a few weeks after diapause emergence found gut microbiotas dominated by core phylotypes (Bosmans, Pozo, Verreth, Crauwels, Wilberts, et al., 2018; Wang et al., 2019); thus it is possible we simply did not examine microbiotas long enough to observe community recovery. It takes approximately 4–5 weeks for bumblebee workers to develop from egg to adult (Heinrich, 2004), so bumblebee queens theoretically have around 1 month for their gut microbiotas to recover if they are to transmit a recovered microbiota to their workers, though their own health would likely benefit from faster recovery (Hammer, Le, Martin, & Moran, 2021). Future studies should serially examine bumblebee queen gut microbiotas from diapause end to eclosion of the first workers to profile changes in community structure during the entire recovery period and determine whether microbiotas consistently return to a pre‐diapause state before worker eclosion.

### Metabolic potential is consistent during and after diapause

We found that at the level of KEGG module categories, metabolic potential is consistent before, during and after diapause (Figures 3 and S7); regardless of treatment, the dominant metabolic categories are amino acid metabolism, carbohydrate metabolism, energy metabolism and metabolism of vitamins and cofactors. This contrasts with hibernating mammal gut microbiotas, where a large metabolic turnover often occurs between active and non‐active seasons as communities shift from being dominated by microbes that metabolize exogenous carbohydrates to those that metabolize host fats and waste (Carey et al., 2013; Sommer et al., 2016; Xiao et al., 2019). We do not observe large taxonomic turnover in bumblebee queen gut microbiotas between active and diapausing treatments—communities are consistently dominated by core bee gut phylotypes (Figure 2). Most core social bee gut phylotypes are sugar fermenters (Hammer, Le, Martin, & Moran, 2021; Kwong & Moran, 2016), thus it is unsurprising that carbohydrate metabolism remains dominant over lipid and nitrogen metabolisms, and that high‐level metabolic potential is consistent through all stages of diapause. However, though metabolic potential (i.e., gene content) is consistent across diapause, it is unlikely the same is true of metabolic activity (i.e., gene expression). Future studies should investigate how transcriptomic, proteomic and/or metabolomic profiles of queen gut microbiotas vary with diapause to further our understanding of how microbiota metabolism changes during this key life stage.

Meanwhile, when examining metabolism at higher resolution, we found that approximately 45% of all observed metabolic modules were enriched in specific treatments (Figures 3c and S8). Here, we were particularly interested in modules that were enriched during diapause but not before or after, as these would provide evidence of metabolic shifts during diapause. However, we do not observe this pattern for any enriched module (Figure S8). Instead, all enriched modules are enriched in pre‐diapause queens, with 11 and 3 modules also enriched in recovery and diapause treatments, respectively. Additionally, some modules that are absent in all individual assemblies within a treatment are present in that treatment's coassembly (Figures S8 and S9). Together, these observations suggest that the enrichment patterns we observe are likely tied to assembly quality. While some enriched modules can be linked to genomic evidence and may truly be enriched in some treatments, such as the enrichment of vitamin and cofactor metabolism in microbiotas with *Schmidhempelia* and *Snodgrassella* (Kwong & Moran, 2016), many others are likely false positives, and these data should be cautiously interpreted.

### Glyphosate does not affect early post‐diapause recovery of queen gut microbiotas

We found that glyphosate has a limited effect on bumblebee queen gut microbial abundance, taxonomic structure and metabolic potential during a 1‐week post‐diapause recovery period. There are no differences between the two recovery treatments in terms of microbial abundance, taxonomic structure, or high‐level metabolic potential (Figures 1a, 2 and 3a). Glyphosate also has no effect on post‐diapause weight, weight gain, or sugar solution consumption (Figures S3C,F and S4).

Notably, *Snodgrassella*, the phylotype that most consistently decreases in abundance after glyphosate exposure (Blot et al., 2019; Castelli et al., 2021; Helander et al., 2023; Motta et al., 2018, 2020; Motta & Moran, 2020, 2023; though see Cullen et al., 2023; Straw et al., 2023), is not detected in queen gut microbiotas from either recovery treatment (Figures 2 and S6). Glyphosate has inconsistent and limited effects on other core social bee microbial phylotypes including *Bifidobacterium* spp., *Bombilactobacillus* spp. and *Lactobacillus* spp. (Blot et al., 2019; Cullen et al., 2023; Helander et al., 2023; Motta et al., 2018, 2020; Motta & Moran, 2020, 2023; Straw et al., 2023), so it is unsurprising that we do not see an effect of glyphosate exposure on queen gut microbial abundance, taxonomic composition, or metabolic potential in our study. As discussed previously, it is possible that *Snodgrassella* is present in recovery treatments at abundances that are not detectable with our methods. An interesting direction for future research would be to conduct a similar recovery experiment but for a longer duration and with more sensitive methods to determine if and when *Snodgrassella* abundance recovers after diapause and if glyphosate exposure impacts this recovery.

## CONCLUSION

Our study is the first to investigate taxonomic and metabolic changes in queen bumblebee gut microbiotas before, during and after diapause. We found that microbial abundance falls by an order of magnitude during diapause and does not recover by 1 week post‐diapause. With this decline in abundance comes taxonomic shifts, while core social bee gut phylotypes dominate gut microbiotas in all treatments, *Schmidhempelia* and *Snodgrassella*, key constituents of pre‐diapause gut microbiotas, are not detected past 2 months of diapause. Despite changes in taxonomic community structure, high‐level metabolic potential is consistent across all treatments. Finally, we found that glyphosate exposure has a limited effect on early post‐diapause queen gut microbiota recovery. These findings, coupled with those of previous studies (Bosmans, Pozo, Verreth, Crauwels, Wäckers, et al., 2018; Bosmans, Pozo, Verreth, Crauwels, Wilberts, et al., 2018; Wang et al., 2019), provide a foundation for future work on the gut microbiotas of diapausing and post‐diapause bumblebee queens. Specifically, we recommend increasing the frequency at which queen gut microbiotas are sampled and the duration of post‐diapause sampling, alongside incorporating methods that allow for characterization of metabolic activity, to obtain a more complete picture of how abundance and community structure shift during and after diapause.

Our results must be interpreted within the limitations of our study. Low microbial abundances caused poor bacterial metagenomic assembly quality for some queen gut microbiotas sampled after the onset of diapause, which resulted in low, uneven sample sizes and high limits of detection for taxa and metabolic pathways. Studies using larger sample sizes and methods to enrich samples for bacterial DNA (Dungan et al., 2023; Ellegaard & Engel, 2019) are needed to verify the patterns we observe. Nevertheless, our study provides valuable insight into how the gut microbiotas of bumblebees, important pollinators in both natural and agro‐ecosystems, change during a key stage in the colony life cycle and furthers our understanding of how host‐associated microbial communities respond to varying environmental conditions.

## METHODS AND MATERIALS

### Queen rearing

We purchased three *B. impatiens* colonies, henceforth referred to as natal colonies, from Biobest (Leamington, Ontario). We removed cotton from colonies upon arrival and kept them in a dark room at 28.5*°*C (±0.09*°*C) and 23.2% relative humidity (±0.6%). We fed natal colonies Biobest BioGluc® syrup and balls of honeybee pollen (Hawkins Honey, Ontario) mixed with water ad libitum. Pollen balls were sterilized with gamma‐irradiation (average dose = 16.43 kGy) before being fed to bees.

We checked natal colonies daily for newly emerged queens and males. When queens emerged, they were weighed (Day 0) and tagged with unique IDs consisting of a letter corresponding to their natal colony (W, R or Y) and a number. After tagging, we released queens back into their natal colonies for 5 days; this period is similar to the duration wild queens spend in their natal colonies (Woodard et al., 2019) and is sufficient for gut microbiota acquisition via conspecific and nest contact (Billiet et al., 2017; Hammer et al., 2023; Meeus et al., 2013). After 5 days (Day 5), we removed queens from their natal colonies and housed them in sterile plastic containers with sibling queens. Whenever males emerged, we removed them from their natal colonies immediately to avoid intra‐colony matings and housed them in sterile plastic containers with sibling males. We kept queens and males in these containers on a 12:12 h light: dark cycle and fed them filter‐sterilized (pore size 0.2 μm) 30% (w/v) sugar solution and sterilized pollen ad libitum.

While we attempted to mate queens with non‐sibling males (see Supporting Information S1), few of our queens mated successfully (<10%), so we ultimately used exclusively unmated queens for our experiment.

### Diapause sampling

We kept queens in their containers until 10 days post‐tagging (Day 10). On Day 10, we once again weighed queens and randomly assigned them to one of five time‐points/treatments (hereafter ‘treatments’): pre‐diapause, 2‐month diapause, 4‐month diapause, diapause‐recovery control or diapause‐recovery with glyphosate. We assigned more queens to diapause and recovery treatments to account for mortality over diapause.

We euthanized queens in the pre‐diapause group (11 total) directly after weighing by freezing at −80°C. We then placed the remaining queens individually in sterile 50‐mL Falcon tubes with holes poked in the lids for ventilation. We placed these vials in an incubator at 14°C for a 2‐day acclimation period. After this, we moved all queens in their Falcon tubes to an incubator set at 4°C for diapause (Yoon et al., 2010, 2013); we maintained humidity at >80% for the duration of diapause.

We sampled 2‐month queens after 2 months of diapause, and 4‐month, recovery control and recovery + glyphosate queens after 4 months of diapause. At sampling time‐points, we removed queens from the incubator and warmed them up to room temperature to verify they were alive. We weighed live queens and then either immediately euthanized them by freezing at −80°C for diapause treatments, or placed them in the recovery experiment. We excluded all queens that died during diapause from further analysis. Across all treatments that underwent diapause, 12 queens were alive in the 2‐month treatment (5 dead), 12 in the 4‐month treatment (12 dead), 11 in the recovery control treatment (9 dead) and 13 in the recovery + glyphosate treatment (10 dead).

### Recovery experiment

After diapause, we placed queens from recovery treatments in individual sterile plastic containers in a dark room at 28.5*°*C (±0.09*°*C) and 23.2% relative humidity (±0.6%) for a 1‐week recovery period. During this time, we provided control queens with sterile 30% (w/v) sugar solution ad libitum, and glyphosate‐treatment queens with 30% sugar solution spiked with glyphosate (PESTANAL®, analytical standard) to a concentration of 0.1 mM ad libitum. We provided all queens with sterile pollen ad libitum. After 1 week, we euthanized all queens by freezing at −80°C.

### DNA extractions and sequencing

We sterilely dissected full guts from all queens, then extracted DNA from a subset of five randomly selected guts per treatment using the QIAGEN DNeasy PowerLyzer PowerSoil Kit (Hilden, Germany) with some modifications (see Supporting Information S1). We did not enrich for prokaryotic cells before extraction as we were interested in observing changes in fungal communities in addition to bacterial and archaeal communities during diapause. We then quantified DNA extracts using a Qubit 2.0 and sent them to Génome Québec for PCR‐free metagenomic shotgun sequencing using the NovaSeq 6000 system; we also sent a negative control, but it did not contain sufficient DNA for sequencing.

### Metagenomic bioinformatics

Here we provide a basic outline of our metagenomic pipeline; for more details see Supporting Information S1.

We first examined raw read quality and trimmed and filtered out low‐quality reads (Andrews, 2019; Chen et al., 2018). After this filtering step, we had a median of 90 million paired‐end reads per sample. We then mapped trimmed reads against a *B. impatiens* genome (NCBI PRJNA61101) and the Kraken2 plant genome database to remove host‐ and pollen‐associated reads (Li, 2013; Sadd et al., 2015; Wood et al., 2019). Afterwards, we had a median of 773,000 paired‐end reads per sample (Table S1).

We assembled filtered reads into contigs (Nurk et al., 2017), performing both individual assemblies by queen and coassemblies by treatment to (1) see if we observe additional taxa in coassemblies and (2) determine if similar patterns in taxonomic and metabolic structure are observed in both assembly types. After, we assessed assembly quality (Ewels et al., 2016; Gurevich et al., 2013) and calculated contig coverages (Danecek et al., 2021; Eren et al., 2020; Li, 2013).

After calculating contig coverage, we assigned high‐level taxonomy to our individual assemblies using the blast+ nt database (Camacho et al., 2009) to determine whether any contigs assigned to eukaryotes (i.e., whether host and pollen contamination was still present). We found that fungal contigs were inconsistently present and that most contigs were assigned to invertebrates, indicating that substantial host contamination remained in our assemblies (Figure S1). Although our initial plan was to examine bacterial and fungal gut communities, due to the inconsistent presence of fungal contigs and low read counts post‐filtering, we decided to limit our microbiota analyses to bacteria. Thus, we selected contigs assigned to ‘bacteria’ from both individual and coassemblies and, after validating that this did not affect bacterial taxonomic profiles (Figure S2), used assemblies containing only bacterial contigs to analyse taxonomic community structure and metabolic potential (see Supporting Information S1 for validation methods and metagenomic assembly statistics).

We used single‐copy core bacterial genes in the Genome Taxonomy Database to determine the taxonomic composition of our assemblies (Eren et al., 2020; Parks et al., 2020); to evaluate the sensitivity of taxonomic profiles to single‐copy gene choice, we compared profiles generated with ribosomal protein S7, the most common single‐copy gene and ribosomal protein S2. We used the KO database from the KEGG to determine the presence of metabolic modules (i.e., metabolic pathways) in our assemblies (Kanehisa et al., 2012; Kanehisa & Goto, 2000; Veseli et al., 2023). We then calculated the relative coverage for each module and identified modules that were consistently present (i.e., enriched) in some treatments and primarily absent in others (Shaiber et al., 2020).

### qPCR for 16S rRNA gene copy number

To estimate microbial abundance, we used qPCR to obtain the total 16S rRNA gene copy number for each sample (see Supporting Information S1). We then multiplied that number by the relative coverage of each taxon present in that sample, correcting for the average number of 16S rRNA gene copies per genome in the given bacterial genus or family according to the *rrn*DB (Stoddard et al., 2015). We used these values to generate distance matrices of gut microbiotas using the vegan package in R and Bray–Curtis dissimilarity (Oksanen et al., 2022).

To avoid confusion when discussing abundance data, we henceforth refer to relative coverage data transformed using total 16S rRNA gene copy counts as ‘gene copy count’ data and untransformed data as ‘relative coverage’ or ‘relative abundance’ data.

### Statistical analyses

Here, we provide an outline of our statistical analyses; for details, see Supporting Information S1.

We conducted all statistical analyses in R v4.3.1 (R Core Team, 2023). We fit all linear models using the ‘lm’ function in the stats package and examined all model assumptions using the performance package (Lüdecke et al., 2021). We performed post‐hoc tests of linear models using the ‘TukeyHSD’ function in the stats package. When we included natal colony in models, we coded it as a fixed factor as it had too few levels to be coded as random. We visualized data using the ggplot2 package (Wickham, 2016).

We used a series of linear models to compare queen weight and weight change across treatments to (1) ensure treatments contained queens of similar physical condition before diapause, (2) ensure queens in the 4‐month diapause and recovery treatments finished diapause in similar condition and (3) determine whether the condition was affected by glyphosate exposure during recovery. We also analysed sugar solution consumption during the recovery experiment using a linear model. For the results of these analyses, see Supporting Information S1.

We analysed how 16S rRNA gene copy counts and the number of paired reads post‐filtering varied across treatments using linear models with treatment and natal colony ID as independent variables. We also tested whether 16S gene copy counts were correlated with the number of reads post‐filtering using Pearson's product–moment correlation.

We calculated the Shannon index for gut microbiotas using the ‘diversity’ function in the vegan package (Oksanen et al., 2022), then used a linear model to determine whether it varied with treatment, with treatment and natal colony ID as independent variables. As data for this model appeared heteroscedastic, we also ran this model with a ranked dependent variable.

We used the ‘adonis2’ function in the vegan package to conduct PERMANOVAs with 9999 permutations to analyse how gut microbial taxonomic community structure varied with treatment (Oksanen et al., 2022). Our response variables were Bray–Curtis dissimilarity matrices generated using vegan, and our independent variables were treatment and natal colony ID. We conducted post‐hoc comparisons using the pairwiseAdonis package (Martinez Arbizu, 2020). We also evaluated homogeneity of group dispersions using the vegan package. To identify which microbial taxa were differentially abundant across treatments, we conducted a ANCOMBC using the ‘ancombc2’ function in the ANCOMBC package (Lin & Das Peddada, 2020). We included treatment and natal colony ID as fixed effects.

We investigated how treatment affected KEGG module category composition (i.e., high‐level metabolic composition) in queen gut microbiotas using the same method we used for taxonomic community structure (i.e., PERMANOVA). To examine which specific KEGG modules (i.e., metabolic pathways) were differentially enriched across treatments, we used the Anvi'o programme anvi‐compute‐metabolic‐enrichment (Eren et al., 2020; Shaiber et al., 2020).

## AUTHOR CONTRIBUTIONS

**Michelle Z. Hotchkiss:** Conceptualization; investigation; funding acquisition; writing – original draft; methodology; validation; visualization; writing – review and editing; formal analysis; data curation. **Jessica R. K. Forrest:** Funding acquisition; writing – review and editing; supervision; resources. **Alexandre J. Poulain:** Funding acquisition; writing – review and editing; supervision; resources.

## CONFLICT OF INTEREST STATEMENT

The authors declare no conflicts of interest.

## Supporting information


**Figure S1.** Relative coverage of taxa in individual metagenomic assemblies based on assignment to the blast+ nt reference library. Only contigs whose top taxonomic assignment had an e‐value ≤1x10^−50^ and a percent identity >90% were used. A) shows relative coverages for invertebrates, vertebrates, plants, fungi, and bacteria, while B) shows relative coverages when examining bacteria and fungi only.
**Figure S2.** Principal coordinates analyses of gut microbiota assemblies using Bray‐Curtis dissimilarities. Points are coloured by assembly ID and shaped by assembly type (i.e., all contigs included or bacterial contigs only). A and C) use taxonomies assigned with ribosomal protein S2, while B and D) use taxonomies assigned with ribosomal protein S7. A and B) display data for individual assemblies while C and D) display data for coassemblies. For both individual and coassemblies, taxonomic community structure does not vary by contig type (PERMANOVA with 9999 permutations; individual assemblies: *F*_1,61_ = 0.03, *p* = 0.99; coassemblies: *F*_1,18_ = 0.003, *p* = 0.99).
**Figure S3.** Box plots of weights and relative weight changes of *Bombus impatiens* queens before, during, and after diapause. A‐C) Weights of queens before diapause, after a four‐month diapause, and after a one‐week recovery period (*n* = 5 per treatment). D‐F) Relative weight changes of queens before diapause, during a four‐month diapause, and during a one‐week recovery period (*n* = 5 per treatment). PD = pre‐diapause, TM = two months, FM = four months, RC = recovery control, RG = recovery + glyphosate. Boxes represent medians and interquartile ranges; the whiskers extend to 1.5 × the interquartile range. Treatment had no effect on weight or relative weight change during any stage of the experiment (all *F* < 0.9, all *p* > 0.4).
**Figure S4.** Volume of sugar solution consumed by *B. impatiens* queens in the preceding 24 hours over the course of a one‐week, post‐diapause recovery. In panel A data are separated by treatment (*n* = 5 per treatment) while in panel B data are separated by natal colony ID (*n* = 3–4 per natal colony). Dots represent means and bars extend to mean ± SE. Solution consumption did not differ by treatment (*F*_1,60_ = 0.84, *p* = 0.36), but did by day (*F*_6,60_ = 3.07, *p* = 0.011) and natal colony ID (*F*_2,60_ = 18.72, *p* < 0.001); consumption was higher on Day 6 than Day 2 (*p* = 0.004) and was lower in queens from natal colony 2 overall (all *p* < 0.001).
**Figure S5.** Box plots of Shannon diversity indices of bumble bee queen gut microbiotas before, during, and after diapause (*n* = 2–5 per treatment). Boxes represent medians and interquartile ranges; the whiskers extend to 1.5 × the interquartile range. Alpha diversity did not vary with treatment, whether using an unranked (*F*_4,9_ = 2.2, *p* = 0.16) or ranked dependent variable (*F*_4,9_ = 2.3, *p* = 0.14).
**Figure S6.** Bumble bee queen gut microbiota taxonomic profiles assigned using ribosomal protein S2. A) Stacked bar plot of relative abundances of microbial phylotypes in bumble bee (*B. impatiens*) queen gut microbiotas faceted by treatment (*n* = 15; n = 1–5 per treatment). B) Stacked bar plot of relative abundances of microbial phylotypes in bumble bee queen gut microbiota coassemblies (coassembled by treatment) (*n* = 5; *n* = 1 per treatment). C) Principal coordinates analysis of bumble bee queen gut microbiotas (*n* = 15; n = 1–5 per treatment) using Bray‐Curtis dissimilarities. Community structure varies with treatment (PERMANOVA: *F*_4,8_ = 3.5, *p* = 0.0001) but not natal colony origin (PERMANOVA: *F*_2,8_ = 1.8, *p* = 0.07).
**Figure S7.** Relative coverages of KEGG module categories (i.e., metabolism types) in queen gut microbiota coassemblies by treatment (*n* = 1 per treatment). Beneath is a bar plot of the number of KEGG modules identified in each coassembly.
**Figure S8.** Relative coverage of enriched KEGG modules in individual queen gut microbiotas, faceted vertically by module category and horizontally by treatment. Note that a module must be present in the majority of microbiotas within a treatment for that module to be considered enriched in the treatment (e.g., arginine biosynthesis is enriched in the pre‐diapause treatment, but not the two‐month diapause or recovery control treatment). Point size corresponds to relative coverage.
**Figure S9.** A list of all enriched KEGG modules in analyses with individual assemblies (Figure S8), and their coverages in queen gut microbiota coassemblies. Plot is faceted by module category. Point size corresponds to relative coverage.

**Table S1.** Host and plant filtering statistics for paired‐read sequence files of bumble bee queen gut microbiotas. Pre‐diap. = pre‐diapause, Two mo. = two‐month diapause, Four mo. = four‐month diapause, Recovery = recovery, and Rec. w/ G. = recovery with glyphosate. # = number, % = percentage.
**Table S2.** Average assembly statistics for metagenomic assemblies performed at the level of individual bumble bee queen gut microbiota, grouped by treatment (*n* = 5 per treatment).
**Table S3.** Assembly statistics for metagenomic coassemblies of bumble bee queen gut microbiotas by treatment (*n* = 1 per treatment).
**Table S4.** Average assembly statistics for metagenomic assemblies performed at the level of individual bumble bee queen gut microbiota after removing all non‐bacterial contigs, grouped by treatment (*n* = 5 per treatment).
**Table S5.** Assembly statistics for metagenomic coassemblies of bumble bee queen gut microbiotas by treatment after removing all non‐bacterial contigs (*n* = 1 per treatment).
**Table S6.** Lengths and GC content of a sample of core social bee gut microbiota reference genomes.

**Data S1.** Supplementary Materials.

## Data Availability

All data and R scripts are available at github.com/michellehotch/Diapause2024. Sequences are available through NCBI Sequence Read Archive PRJNA1066198. Six raw sequence files became corrupted during storage after analysis was complete, so we provide host‐filtered sequence files for all samples in addition to all uncorrupted raw sequence files.
