# Supplementary material for: Changes in bumblebee queen gut microbiotas during and after overwintering diapause
Source: Insect Mol Biol. 2024 Aug 22;34(1):136–50. doi: 10.1111/imb.12957 (PMC11705525; doi:10.1111/imb.12957)
Supplement: Supplementary file 1 — Figure S1. Relative coverage of taxa in individual metagenomic assemblies based on assignment to the blast+ nt reference library. Only contigs whose top taxonomic assignment had an e‐value ≤1x10^−50^ and a percent identity >90% were used. A) shows relative coverages for invertebrates, vertebrates, plants, fungi, and bacteria, while B) shows relative coverages when examining bacteria and fungi only. Figure S2. Principal coordinates analyses of gut microbiota assemblies using Bray‐Curtis dissimilarities. Points are coloured by assembly ID and shaped by assembly type (i.e., all contigs included or bacterial contigs only). A and C) use taxonomies assigned with ribosomal protein S2, while B and D) use taxonomies assigned with ribosomal protein S7. A and B) display data for individual assemblies while C and D) display data for coassemblies. For both individual and coassemblies, taxonomic community structure does not vary by contig type (PERMANOVA with 9999 permutations; individual assemblies: F 1,61 = 0.03, p = 0.99; coassemblies: F 1,18 = 0.003, p = 0.99). Figure S3. Box plots of weights and relative weight changes of Bombus impatiens queens before, during, and after diapause. A‐C) Weights of queens before diapause, after a four‐month diapause, and after a one‐week recovery period (n = 5 per treatment). D‐F) Relative weight changes of queens before diapause, during a four‐month diapause, and during a one‐week recovery period (n = 5 per treatment). PD = pre‐diapause, TM = two months, FM = four months, RC = recovery control, RG = recovery + glyphosate. Boxes represent medians and interquartile ranges; the whiskers extend to 1.5 × the interquartile range. Treatment had no effect on weight or relative weight change during any stage of the experiment (all F < 0.9, all p > 0.4). Figure S4. Volume of sugar solution consumed by B. impatiens queens in the preceding 24 hours over the course of a one‐week, post‐diapause recovery. In panel A data are separated by treatment (n = 5 per [file IMB-34-136-s003.docx]

**
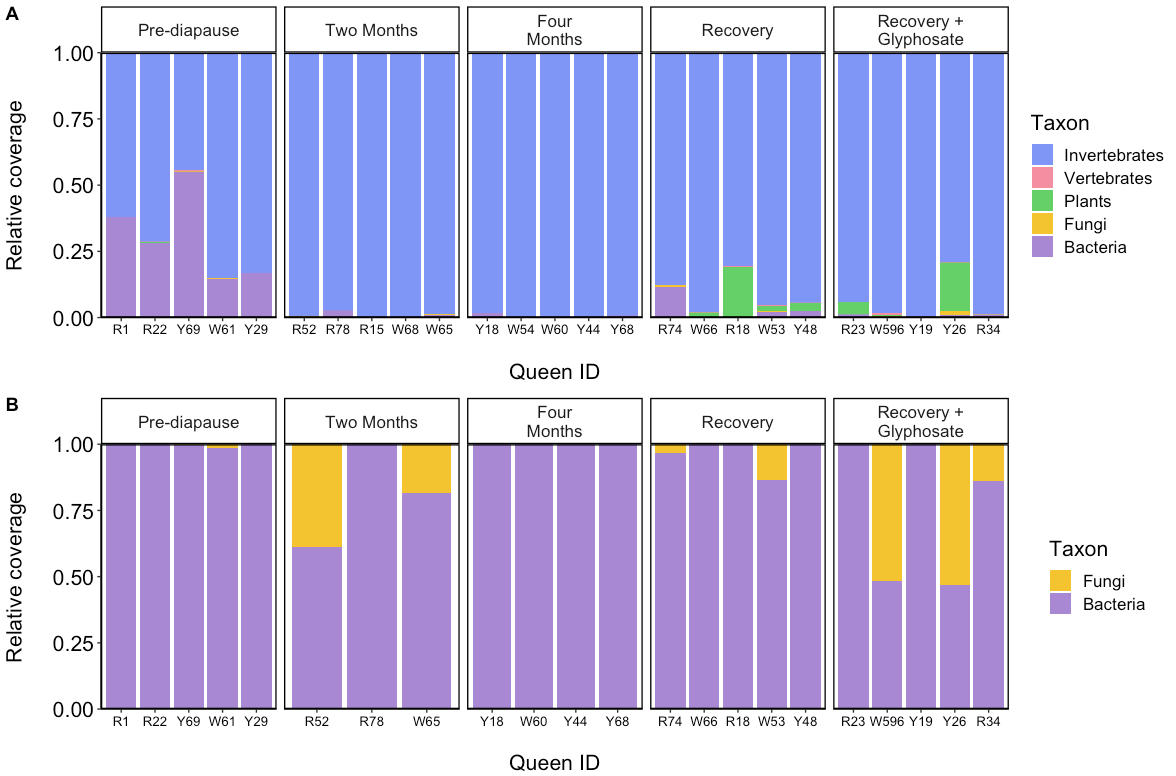
Figure S1:** Relative coverage of taxa in individual metagenomic assemblies based on assignment to the blast+ nt reference library. Only contigs whose top taxonomic assignment had an e-value ≤1x10^-50^ and a percent identity >90% were used. A) shows relative coverages for invertebrates, vertebrates, plants, fungi, and bacteria, while B) shows relative coverages when examining bacteria and fungi only.


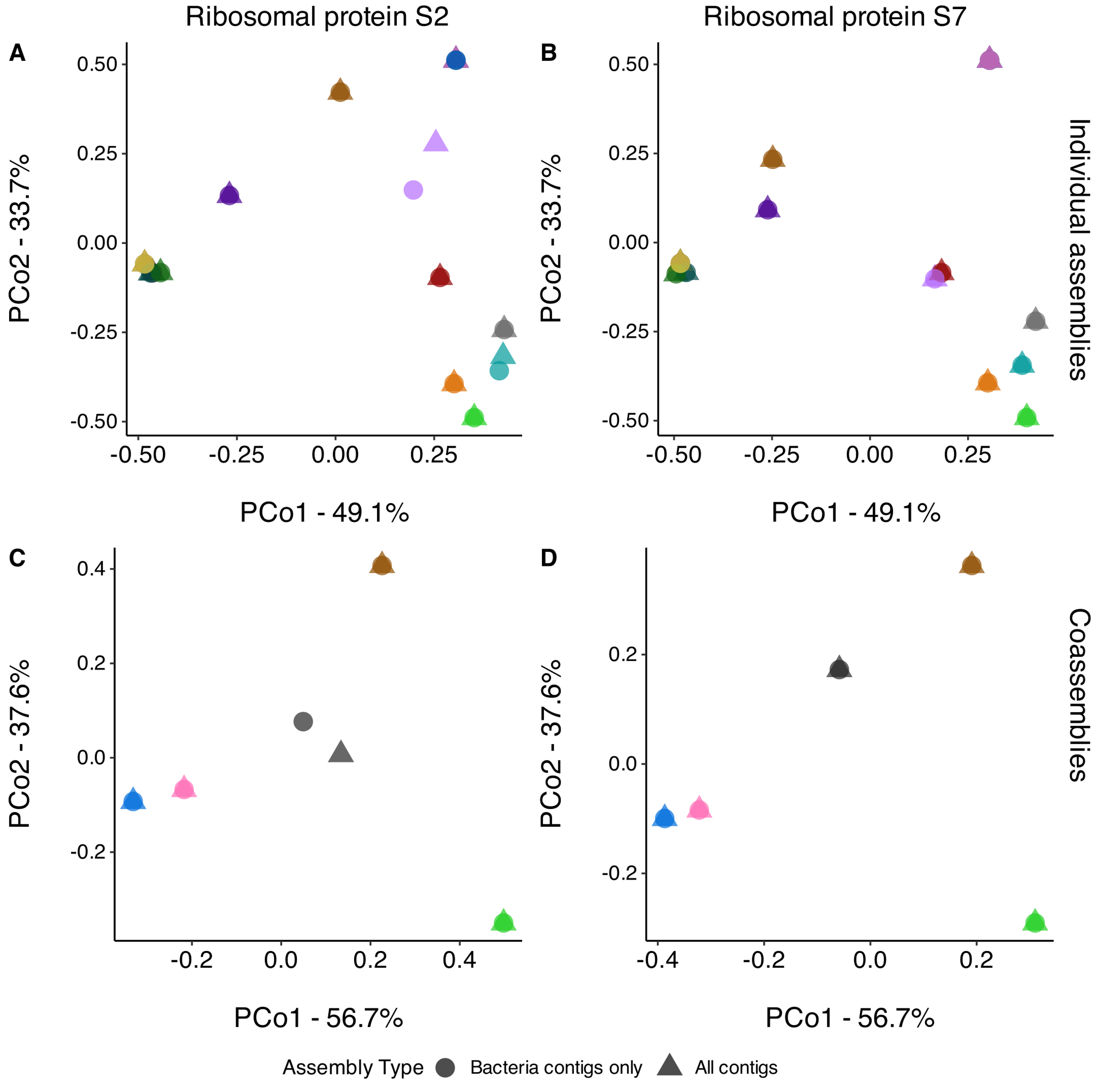


**Figure S2:** Principal coordinates analyses of gut microbiota assemblies using Bray-Curtis dissimilarities. Points are coloured by assembly ID and shaped by assembly type (i.e., all contigs included or bacterial contigs only). A and C) use taxonomies assigned with ribosomal protein S2, while B and D) use taxonomies assigned with ribosomal protein S7. A and B) display data for individual assemblies while C and D) display data for coassemblies. For both individual and coassemblies, taxonomic community structure does not vary by contig type (PERMANOVA with 9,999 permutations; individual assemblies: *F*_1,61_ = 0.03, *p* = 0.99; coassemblies: *F*_1,18_ = 0.003, *p* = 0.99).

**
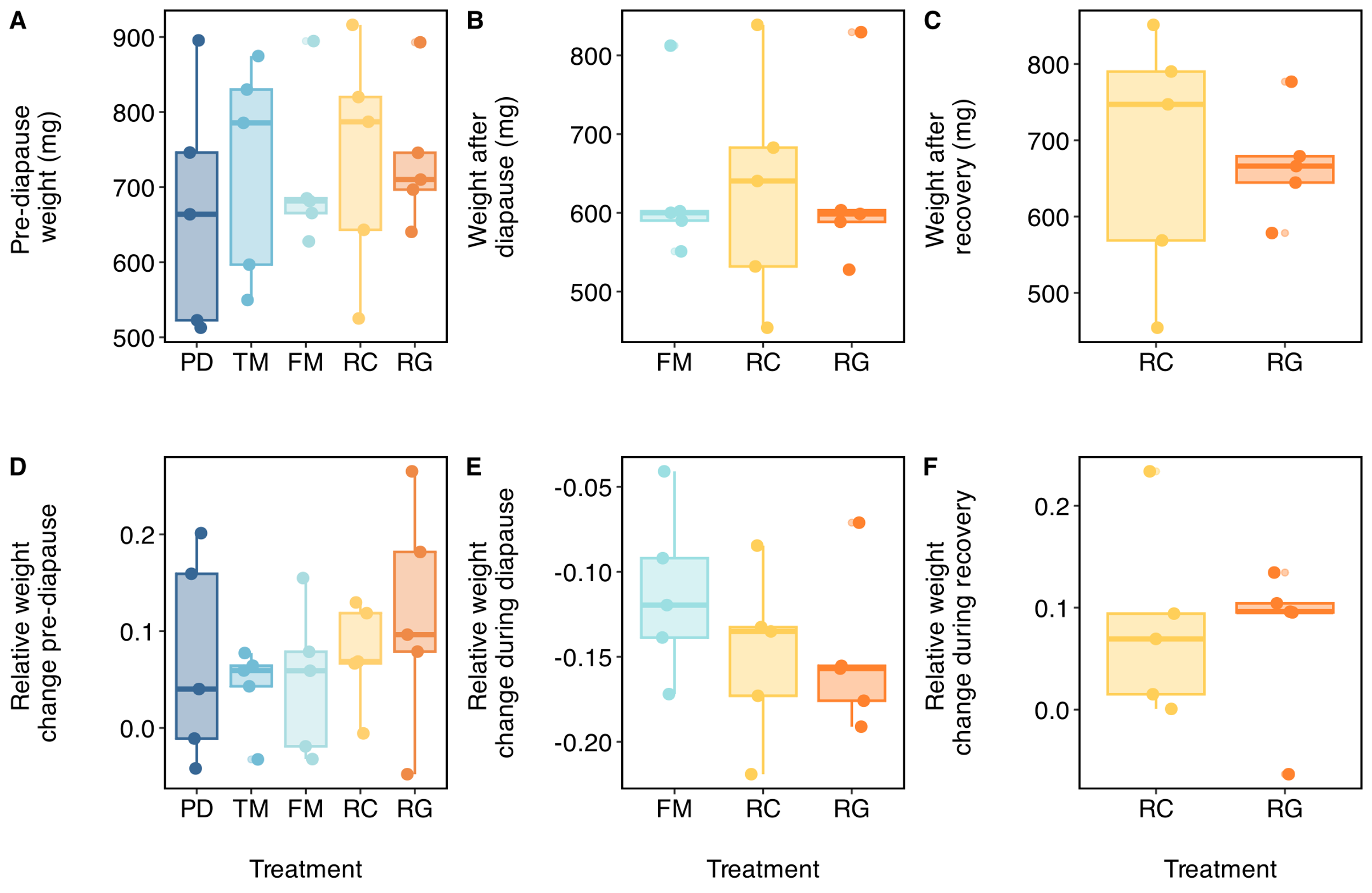
**

**Figure S3**: Box plots of weights and relative weight changes of *Bombus impatiens* queens before, during, and after diapause. A-C) Weights of queens before diapause, after a four-month diapause, and after a one-week recovery period (n=5 per treatment). D-F) Relative weight changes of queens before diapause, during a four-month diapause, and during a one-week recovery period (n=5 per treatment). PD = pre-diapause, TM = two months, FM = four months, RC = recovery control, RG = recovery + glyphosate. Boxes represent medians and interquartile ranges; the whiskers extend to 1.5 × the interquartile range. Treatment had no effect on weight or relative weight change during any stage of the experiment (all *F* < 0.9, all *p* > 0.4).


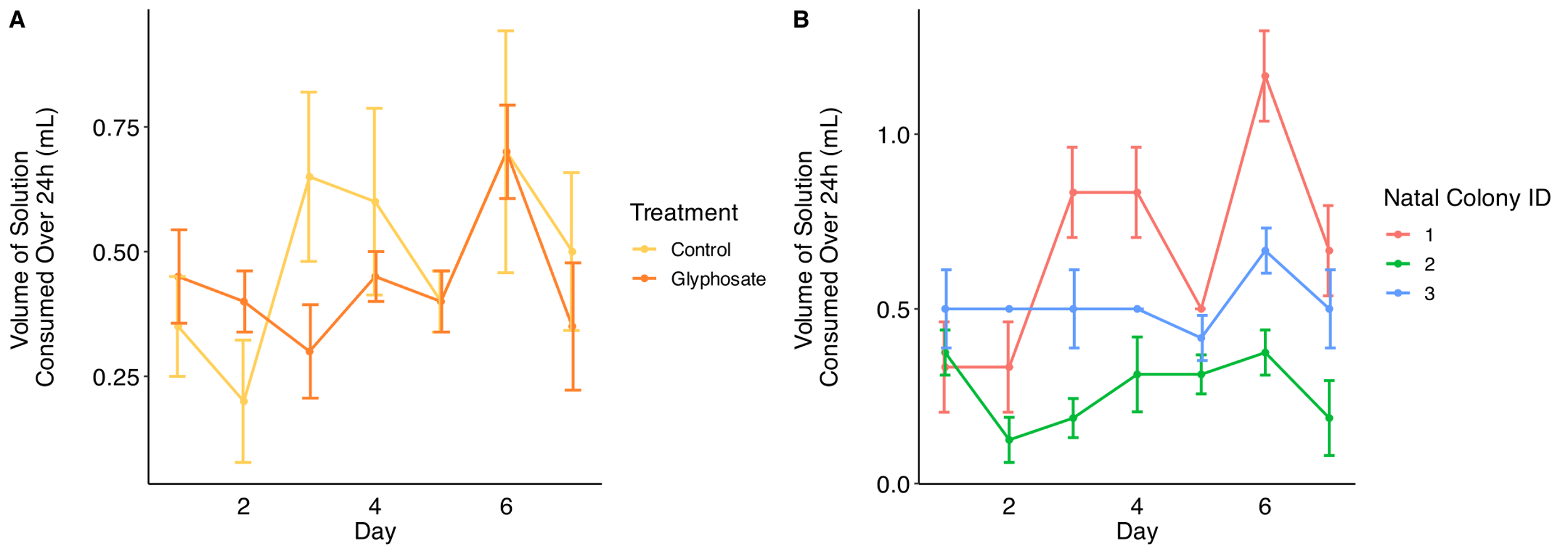


**Figure S4:** Volume of sugar solution consumed by *B. impatiens* queens in the preceding 24 hours over the course of a one-week, post-diapause recovery. In panel A data are separated by treatment (n = 5 per treatment) while in panel B data are separated by natal colony ID (n = 3–4 per natal colony). Dots represent means and bars extend to mean±SE. Solution consumption did not differ by treatment (*F*_1,60_ = 0.84, *p* = 0.36), but did by day (*F*_6,60_ = 3.07, *p* = 0.011) and natal colony ID (*F*_2,60_ = 18.72, *p* < 0.001); consumption was higher on Day 6 than Day 2 (*p* = 0.004) and was lower in queens from natal colony 2 overall (all *p* < 0.001).


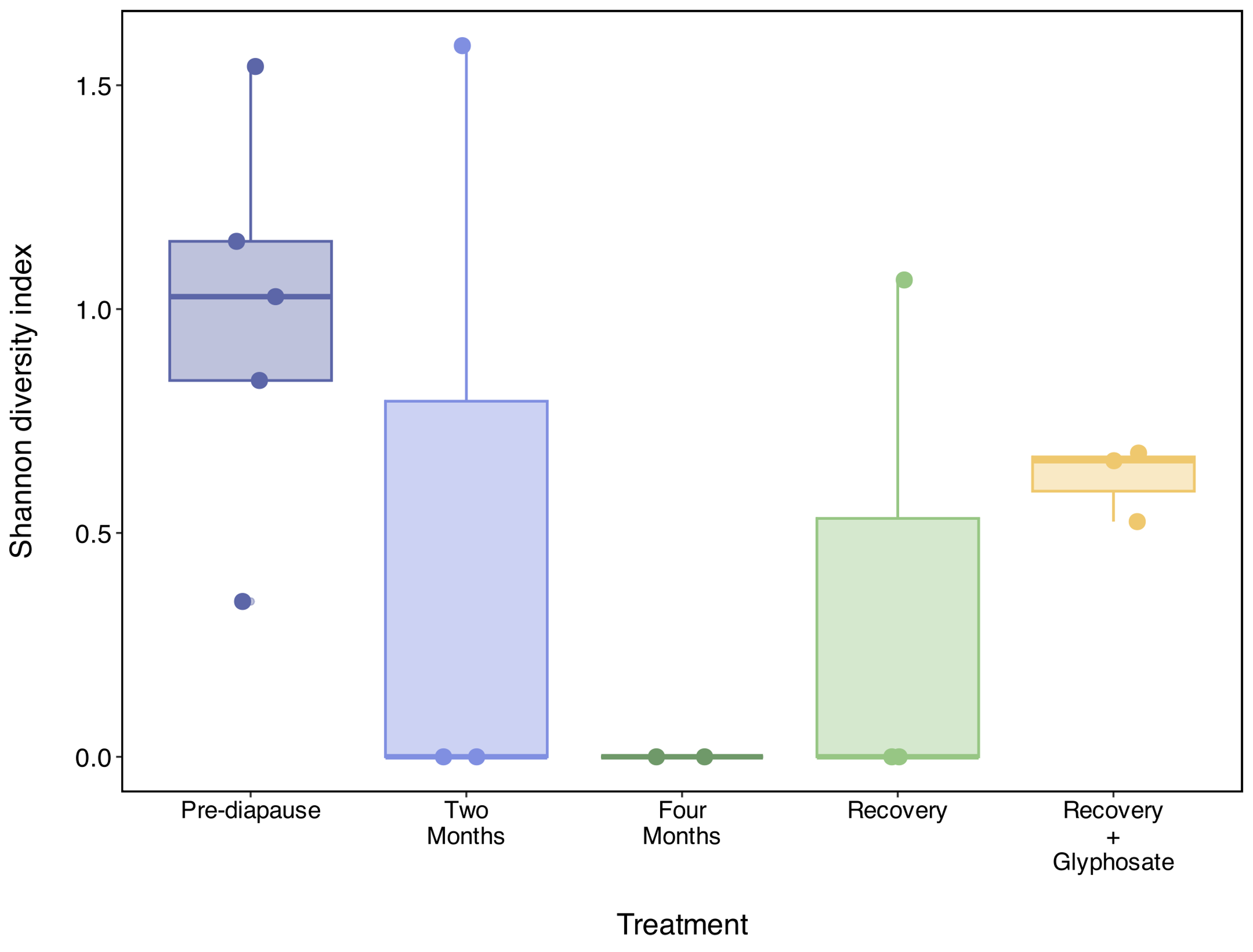


**Figure S5:** Box plots of Shannon diversity indices of bumble bee queen gut microbiotas before, during, and after diapause (n = 2-5 per treatment). Boxes represent medians and interquartile ranges; the whiskers extend to 1.5 × the interquartile range. Alpha diversity did not vary with treatment, whether using an unranked (*F*_4,9_ = 2.2, *p* = 0.16) or ranked dependent variable (*F*_4,9_ = 2.3, *p* = 0.14).


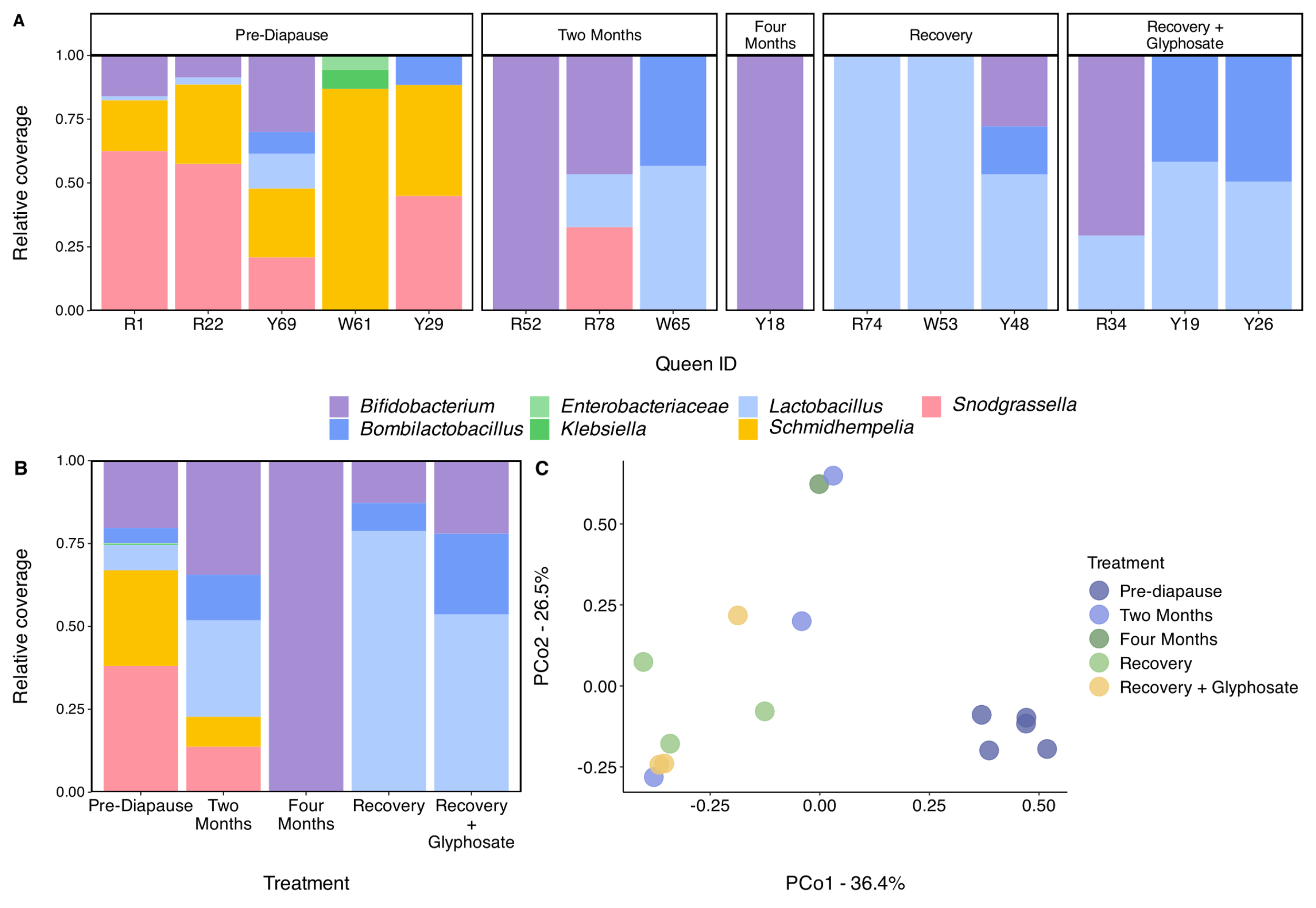


**Figure S6**: Bumble bee queen gut microbiota taxonomic profiles assigned using ribosomal protein S2. A) Stacked bar plot of relative abundances of microbial phylotypes in bumble bee (*B. impatiens*) queen gut microbiotas faceted by treatment (n = 15; n = 1-5 per treatment). B) Stacked bar plot of relative abundances of microbial phylotypes in bumble bee queen gut microbiota coassemblies (coassembled by treatment) (n = 5; n = 1 per treatment). C) Principal coordinates analysis of bumble bee queen gut microbiotas (n = 15; n = 1-5 per treatment) using Bray-Curtis dissimilarities. Community structure varies with treatment (PERMANOVA: *F*_4,8_ = 3.5, *p* = 0.0001) but not natal colony origin (PERMANOVA: *F*_2,8_ = 1.8, *p* = 0.07).


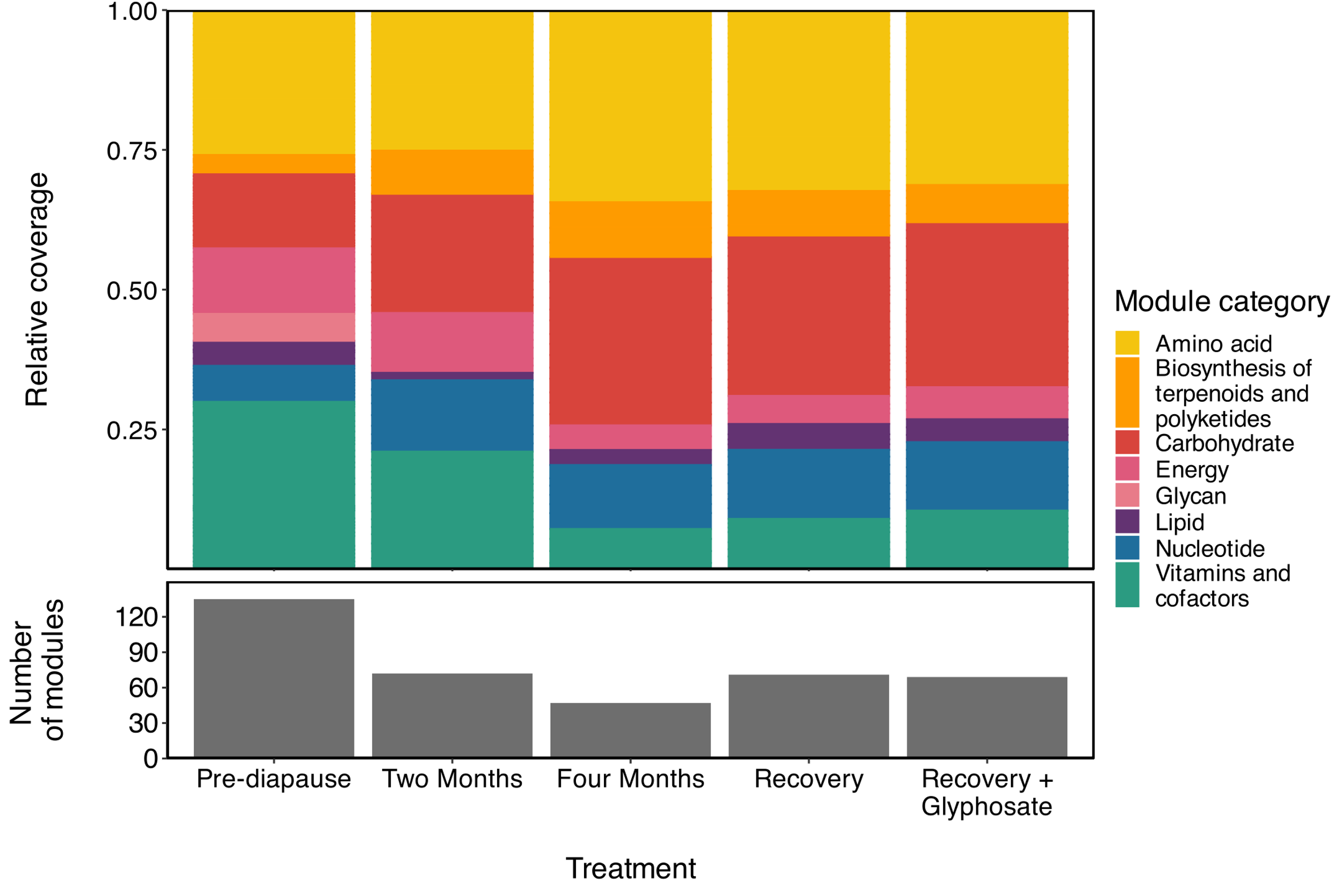


**Figure S7**: Relative coverages of KEGG module categories (i.e., metabolism types) in queen gut microbiota coassemblies by treatment (n = 1 per treatment). Beneath is a bar plot of the number of KEGG modules identified in each coassembly.


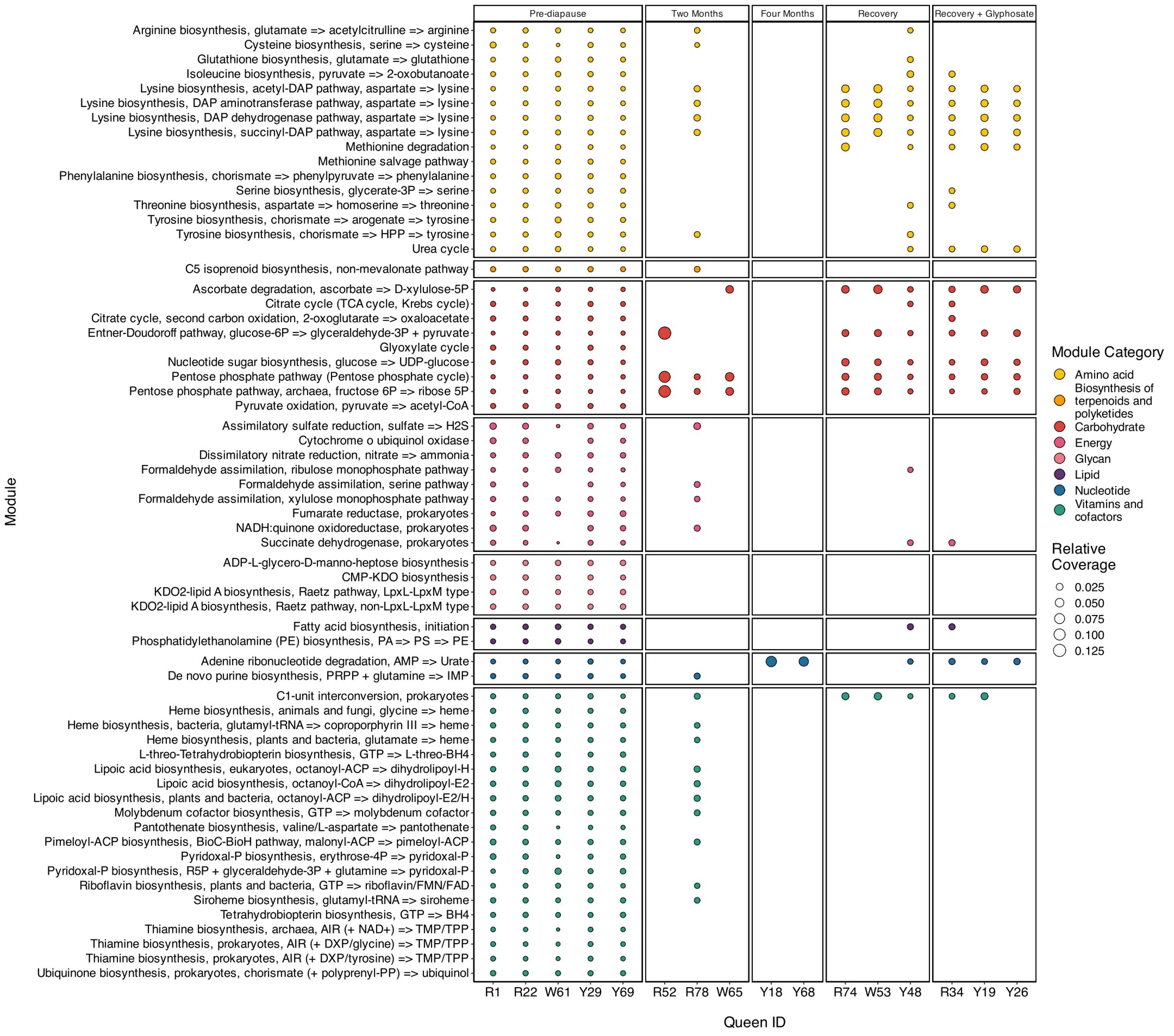


**Figure S8:** Relative coverage of enriched KEGG modules in individual queen gut microbiotas, faceted vertically by module category and horizontally by treatment. Note that a module must be present in the majority of microbiotas within a treatment for that module to be considered enriched in the treatment (e.g., arginine biosynthesis is enriched in the pre-diapause treatment, but not the two-month diapause or recovery control treatment). Point size corresponds to relative coverage.


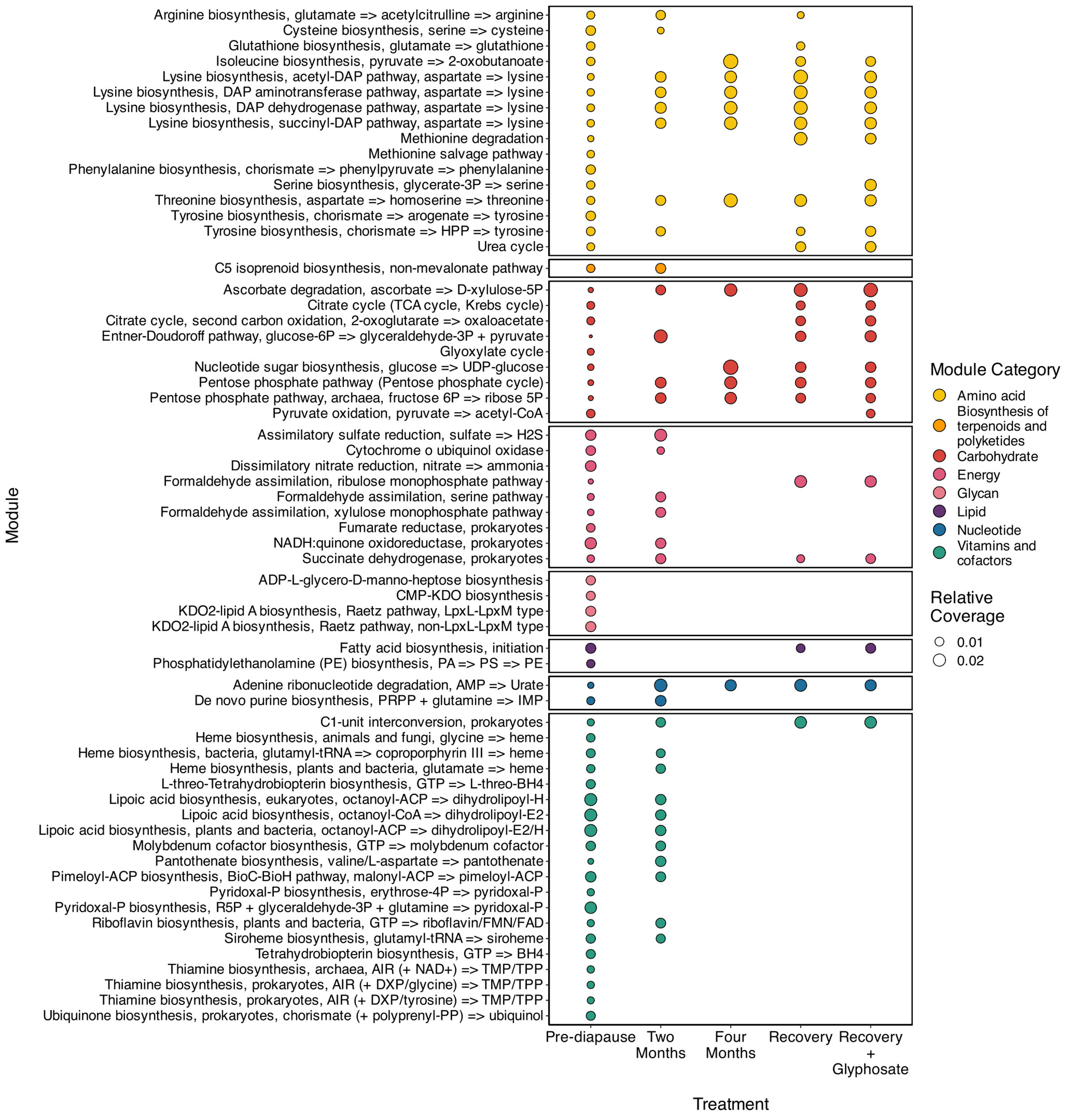


**Figure S9:** A list of all enriched KEGG modules in analyses with individual assemblies (Fig. S8), and their coverages in queen gut microbiota coassemblies. Plot is faceted by module category. Point size corresponds to relative coverage.
