# Supplementary material for: Changes in bumblebee queen gut microbiotas during and after overwintering diapause
Source: Insect Mol Biol. 2024 Aug 22;34(1):136–50. doi: 10.1111/imb.12957 (PMC11705525; doi:10.1111/imb.12957)
Supplement: Supplementary file 2 — Table S1. Host and plant filtering statistics for paired‐read sequence files of bumble bee queen gut microbiotas. Pre‐diap. = pre‐diapause, Two mo. = two‐month diapause, Four mo. = four‐month diapause, Recovery = recovery, and Rec. w/ G. = recovery with glyphosate. # = number, % = percentage. Table S2. Average assembly statistics for metagenomic assemblies performed at the level of individual bumble bee queen gut microbiota, grouped by treatment (n = 5 per treatment). Table S3. Assembly statistics for metagenomic coassemblies of bumble bee queen gut microbiotas by treatment (n = 1 per treatment). Table S4. Average assembly statistics for metagenomic assemblies performed at the level of individual bumble bee queen gut microbiota after removing all non‐bacterial contigs, grouped by treatment (n = 5 per treatment). Table S5. Assembly statistics for metagenomic coassemblies of bumble bee queen gut microbiotas by treatment after removing all non‐bacterial contigs (n = 1 per treatment). Table S6. Lengths and GC content of a sample of core social bee gut microbiota reference genomes. [file IMB-34-136-s002.docx]

**Table S1**: Host and plant filtering statistics for paired-read sequence files of bumble bee queen gut microbiotas. Pre-diap. = pre-diapause, Two mo. = two-month diapause, Four mo. = four-month diapause, Recovery = recovery, and Rec. w/ G. = recovery with glyphosate. # = number, % = percentage.

| Queen ID | Treatment | # of raw reads | # reads post-quality filtering | # reads post-host filtering | # reads post-plant filtering | % raw reads remaining post-filtering |
| --- | --- | --- | --- | --- | --- | --- |
| R15 | Two mo. | 99,265,244 | 97,881,894 | 1,102,202 | 836,359 | 0.84 |
| R18 | Recovery | 81,369,165 | 80,340,533 | 1,467,402 | 806,605 | 0.99 |
| R23 | Rec. w/ G. | 81,928,241 | 80,834,806 | 869,196 | 615,470 | 0.75 |
| R34 | Rec. w/ G. | 85,897,067 | 84,608,912 | 1,048,842 | 811,919 | 0.95 |
| W53 | Recovery | 79,306,396 | 78,347,287 | 803,532 | 578,302 | 0.73 |
| W54 | Four mo. | 117,471,111 | 115,614,362 | 999,084 | 773,083 | 0.66 |
| W596 | Rec. w/ G. | 77,479,236 | 76,498,166 | 716,331 | 536,223 | 0.69 |
| W60 | Four mo. | 101,365,555 | 99,706,802 | 782,818 | 600,210 | 0.59 |
| W61 | Pre-diap. | 91,551,732 | 90,273,670 | 1,008,462 | 807,523 | 0.88 |
| W65 | Two mo. | 97,329,919 | 96,024,520 | 930,569 | 717,614 | 0.74 |
| W66 | Recovery | 74,760,197 | 73,697,421 | 1,006,384 | 516,600 | 0.69 |
| W68 | Two mo. | 94,455,902 | 92,875,992 | 736,109 | 568,683 | 0.60 |
| R1 | Pre-diap. | 101,249,083 | 99,725,111 | 2,523,382 | 2,210,791 | 2.18 |
| Y18 | Four mo. | 89,366,170 | 87,886,225 | 647,962 | 508,723 | 0.57 |
| Y19 | Rec. w/ G. | 77,281,232 | 76,214,734 | 669,699 | 525,827 | 0.68 |
| R22 | Pre-diap. | 76,834,433 | 75,758,902 | 1,416,804 | 1,219,545 | 1.59 |
| Y26 | Rec. w/ G. | 83,236,115 | 81,987,870 | 1,406,222 | 651,534 | 0.78 |
| Y29 | Pre-diap. | 94,004,688 | 92,651,450 | 1,258,235 | 1,053,440 | 1.12 |
| R44 | Four mo. | 115,293,837 | 113,487,677 | 1,369,320 | 1,048,501 | 0.91 |
| Y48 | Recovery | 99,132,233 | 97,497,367 | 1,011,681 | 770,207 | 0.78 |
| Y52 | Two mo. | 88,520,320 | 86,964,863 | 1,037,977 | 789,496 | 0.89 |
| Y68 | Four mo. | 102,356,290 | 100,970,866 | 824,777 | 642,682 | 0.63 |
| Y69 | Pre-diap. | 99,955,029 | 98,573,968 | 3,852,131 | 3,601,528 | 3.60 |
| R74 | Recovery | 78,401,255 | 77,346,140 | 1,074,890 | 869,212 | 1.11 |
| R78 | Two mo. | 119,875,442 | 117,772,307 | 1,466,198 | 1,131,511 | 0.94 |

**Table S2:** Average assembly statistics for metagenomic assemblies performed at the level of individual bumble bee queen gut microbiota, grouped by treatment (n=5 per treatment).

| Treatment | Mean num. of contigs | Min num. of contigs | Max num. of contigs | Mean largest contig (Kbp) | Mean assem. length (Mbp) | Mean N50 (Kbp) | Mean L50 (K) |
| --- | --- | --- | --- | --- | --- | --- | --- |
| Pre-diapause | 8,907.6 | 4,172 | 18,051 | 417.36 | 9.8 | 56.1 | 0.06 |
| Two-month diapause | 5,384.2 | 1,312 | 13,801 | 5.14 | 1 | 0.92 | 0.28 |
| Four-month diapause | 4,266.2 | 1,676 | 6,389 | 3.46 | 0.42 | 0.8 | 0.16 |
| Recovery | 14,713.2 | 3,996 | 31,382 | 97.72 | 2.42 | 17.8 | 0.16 |
| Recovery w/ glyphosate | 12,702.8 | 4,950 | 27,058 | 24.52 | 2.28 | 1.94 | 0.24 |

**Table S3:** Assembly statistics for metagenomic coassemblies of bumble bee queen gut microbiotas by treatment (n=1 per treatment).

| Treatment | Number of contigs | Largest contig (Kbp) | Assembly length (Mbp) | N50 (Kbp) | L50 (K) |
| --- | --- | --- | --- | --- | --- |
| Pre-diapause | 25,003 | 838.5 | 26.2 | 50 | 1.5 |
| Two-month diapause | 19,134 | 9.3 | 5.3 | 1.1 | 0.7 |
| Four-month diapause | 14,072 | 6.9 | 2.2 | 1.2 | 0.8 |
| Recovery | 62,228 | 185.1 | 8.3 | 7.5 | 1.4 |
| Recovery w/ glyphosate | 49,714 | 105.5 | 7.1 | 6.6 | 1.7 |

| Treatment | Mean num. of contigs | Min num. of contigs | Max num. of contigs | Mean largest contig (Kbp) | Mean assem. length (Mbp) | Mean N50 (Kbp) | Mean L50 (K) |
| --- | --- | --- | --- | --- | --- | --- | --- |
| Pre-diapause | 2,299.8 | 963 | 4,934 | 417.36 | 8.2 | 75.42 | 0.02 |
| Two-month diapause | 885.2 | 4 | 2,189 | 3.92 | 0.48 | 1.16 | 0.12 |
| Four-month diapause | 290.2 | 4 | 797 | 1.84 | 0.1 | 1.08 | 0.04 |
| Recovery | 437.2 | 8 | 1,881 | 96.1 | 1.84 | 19.4 | 0.02 |
| Recovery w/ glyphosate | 1,025.2 | 21 | 2,061 | 23.56 | 1.64 | 2.6 | 0.12 |

**Table S4:** Average assembly statistics for metagenomic assemblies performed at the level of individual bumble bee queen gut microbiota after removing all non-bacterial contigs, grouped by treatment (n=5 per treatment).

**Table S5:** Assembly statistics for metagenomic coassemblies of bumble bee queen gut microbiotas by treatment after removing all non-bacterial contigs (n=1 per treatment).

| Treatment | Number of contigs | Largest contig (Kbp) | Assembly length (Mbp) | N50 (Kbp) | L50 (K) |
| --- | --- | --- | --- | --- | --- |
| Pre-diapause | 6,554 | 838.5 | 20.8 | 104.1 | 0 |
| Two-month diapause | 4,467 | 9.3 | 2.9 | 1.2 | 0.7 |
| Four-month diapause | 1,282 | 6.9 | 1 | 1.6 | 0.2 |
| Recovery | 1,555 | 185.1 | 6.1 | 25.3 | 0 |
| Recovery w/ glyphosate | 1,292 | 105.5 | 5.2 | 12.3 | 0.1 |

**Table S6:** Lengths and GC content of a sample of core social bee gut microbiota reference genomes.

| Genus | Species | NCBI Assembly ID | Length (Mbp) | GC content (%) |
| --- | --- | --- | --- | --- |
| *Bifidobacterium* | *actinocoloniiforme* | ASM126339v1 | 1.8 | 62.5 |
|  | *asteroides* | ASM30421v1 | 2.2 | 60.0 |
|  | *bohemicum* | IMG-taxon 2616644833 | 2.0 | 57.5 |
|  | *bombi* | DSM-19703 | 1.9 | 56.0 |
|  | *commune* | IMG-taxon 2616644832 | 1.6 | 54.0 |
| *Bombilactobacillus* | *apium* | ASM1338514v1 | 1.7 | 40.0 |
|  | *bombi* | ASM352296v1 | 1.8 | 34.5 |
|  | *folatiphilus* | ASM2338026v1 | 1.6 | 38.5 |
|  | *mellifer* | ASM97079v1 | 1.8 | 39.5 |
|  | *mellis* | ASM96724v1 | 1.8 | 36.0 |
|  | *thymidiniphilus* | ASM2338024v1 | 1.5 | 36.5 |
| *Lactobacillus* | *apis* | ASM315093v1 | 1.7 | 37.5 |
|  | *bombicola* | ASM291691v1 | 1.7 | 34.5 |
|  | *helsingborgensis* | ASM317369v1 | 1.9 | 37.0 |
|  | *helveticus* | ASM305308v1 | 2.1 | 36.5 |
|  | *kullabergensis* | ASM315102v1 | 2.0 | 36.0 |
|  | *melliventris* | ASM97077v1 | 2.1 | 36.0 |
|  | *panisapium* | ASM1946926v1 | 2.2 | 38.0 |
| *Candidatus* Schmidhempelia | *bombi* | BiG_1.2 | 2.2 | 36.5 |
| *Snodgrassella* | *alvi* | ASM60000v1 | 2.5 | 41.5 |
|  | *communis* | LMG 28360 | 2.3 | 43.5 |
|  | *gandavensis* | LMG 30236 | 2.5 | 44.0 |
