# Supplementary material for: Changes in bumblebee queen gut microbiotas during and after overwintering diapause
Source: Insect Mol Biol. 2024 Aug 22;34(1):136–50. doi: 10.1111/imb.12957 (PMC11705525; doi:10.1111/imb.12957)
Supplement: Supplementary file 3 — Data S1. Supplementary Materials. [file IMB-34-136-s001.docx]

## ­­­­Changes in bumblebee queen gut microbiotas during and after diapause

Michelle Z. Hotchkiss, Jessica R. K. Forrest, and Alexandre J. Poulain

## Supplementary Materials

## Methods

### Queen Mating

Two days after each queen’s removal from her natal colony (Day 7), we attempted to mate her with a male from a different colony. We released two to four queens and at least twice the number of males into a flight cage (40 cm x 40 cm x 60 cm) under bright light and observed bees for mating events. However, few of our queens mated successfully (<10%), and so we ultimately used exclusively unmated queens for our experiment.

### Dissections

We dissected all queens in a sterile field using a dissecting microscope. We first soaked queens for three minutes in a 1% bleach solution to kill any cuticular microbes­ then rinsed them three times in sterile water (Engel *et al.*, 2013). We then moved queens to a dissection dish containing sterile Ringer’s solution (2.16 g NaCl, 0.051 g CaCl2, 0.111 g KCl in 300 mL of water) and removed each queen’s entire gut. We froze guts at -20˚C until DNA extractions. For each new dissection, we used fresh Ringer’s solution and a new sterile dissection dish; we also cleaned the microscope with 70% ethanol and flame-sterilized all dissection tools between dissections.

### DNA Extractions and Sequencing

We extracted DNA from a subset of five, randomly-selected queen guts per treatment using the QIAGEN DNeasy PowerLyzer PowerSoil Kit (Hilden, Germany), following protocol with the following modifications: 1) gut samples were masticated using a pestle before extraction, 2) after 60 µL of solution C1 was added and samples were vortexed, samples were incubated in a water bath for 10 minutes at 65˚C, 3) all centrifugation steps were conducted at 13,000 x *g*, and 4) solution C6 sat on the membrane for 5 minutes before the samples were centrifuged for elution; we did not enrich for prokaryotic cells before extraction as we were interested in observing changes in fungal communities in addition to bacterial and archaeal communities during diapause. We quantified DNA using a Qubit 2.0 with the Invitrogen Qubit dsDNA HS Assay Kit following protocol with the modification that the DNA was incubated in the dye/buffer mixture for five minutes. We sent extracted DNA and a negative control to Génome Québec for PCR-free metagenomic shotgun sequencing using the NovaSeq 6000 system; the negative control did not contain sufficient DNA for sequencing.

### Metagenomic Bioinformatics

We examined the quality of raw reads using FastQC v0.11.9 (Andrews, 2019), and trimmed and filtered out low-quality reads using fastp v0.23.2 with default settings (Chen *et al.*, 2018). After this filtering step, we had a median of 90 million paired-end reads per sample (range: 73.6 million – 117.8 million). We then used the bwa-mem command in bwa v0.7.17 to map trimmed reads against a *B. impatiens* genome (NCBI PRJNA61101) to remove host reads (Li, 2013; Sadd *et al.*, 2015), and used Kraken2 v2.1.2 to map host-filtered reads against the Kraken2 standard plant reference library to remove pollen-associated sequences (Wood *et al.*, 2019). After filtering out reads that mapped to the host genome and Kraken2 plant reference library, we had a median of 773,000 paired-end reads per sample (range: 508,723 – 3,601,528); >99% of paired reads were lost at the host-filtering step (Table S1).

We assembled fully filtered reads using metaSPAdes v3.15.4 with paired-end library type and default settings, including k: 21, 33, 55 (Nurk *et al.*, 2017). We performed individual assemblies by queen and coassemblies by treatment to 1) see if we observe additional taxa in coassemblies and 2) determine if similar patterns in taxonomic and metabolic structure are observed in both assembly types. We assessed assembly quality using quast v5.0.2 and multiqc v1.9 (Gurevich *et al.*, 2013; Ewels *et al.*, 2016). We then used the anvi-script-reformat-fasta program from Anvi’o v7.1-dev to reformat the assemblies for further analysis and remove contigs less than 300 bp (Eren *et al.*, 2020; Shell & Rehan, 2022). For each assembly, we generated contig databases in Anvi’o using anvi-gen-contigs-database program (Eren *et al.*, 2020). We calculated contig coverages using bwa-mem to map filtered reads against contigs, samtools v1.17 to convert mapping output to the bam file type, and Anvi’o’s anvi-init-bam, anvi-profile, and anvi-export-splits-and-coverages to calculate and extract the coverage for each contig for each assembly (Li, 2013; Eren *et al.*, 2020; Danecek *et al.*, 2021).

After calculating the coverage for each contig in each assembly, we wanted to obtain a high-level taxonomic overview of our individual assemblies to determine whether any contigs were being assigned to eukaryotes (i.e., whether host and pollen contamination was still present). To accomplish this, we used blast+ v2.14.1 to assign taxonomy to all contigs in individual assemblies based on alignment against the blast+ nt database (Camacho *et al.*, 2009). We selected the top taxonomic hit for each contig in each assembly, filtered for taxonomic hits with e-values < 1x10^-50^ and percent identities >90%, and then calculated the mean coverage for each of six high-level taxa of interest – bacteria, fungi, plants, invertebrates, and vertebrates. We found that most contigs were assigned to invertebrates, indicating that substantial host contamination remained in our assemblies (Fig. S1A), and that fungal contigs were inconsistently present in individual assemblies (Fig. S1B). Although our initial plan was to examine taxonomic and metabolic changes in bacterial and fungal communities in bumblebee queen gut microbiotas, due to the inconsistent presence of fungal contigs, the complexity of eukaryotic genes and genomes, and low number of reads post-host and plant filtering, we decided to limit our analysis to bacterial communities.

To eliminate eukaryotic contigs from our assemblies, we used the “dplyr” package (v1.1.2) in R (v4.3.1) (R Core Team, 2023; Wickham *et al.*, 2023) to generate a list of all contigs assigned to the kingdom “bacteria” by blast+ for each individual assembly; we did not filter assignments by e-value or percent identity at this step. We then used those lists to subset only bacteria-assigned contigs from each of the individual assemblies. We also repeated the blast+ assignment and bacteria filtering step for the coassemblies.

At this point in our analysis, we had four types of assembly: 1) individual queen assemblies that contained all contigs 300 bp or longer (full individual assemblies), 2) individual queen assemblies that contained all contigs 300 bp or longer assigned to bacteria in blast+ (bacterial individual assemblies), 3) coassemblies by treatment that included all contigs 300 bp or longer (full coassemblies), and 4) coassemblies by treatment that included all contigs 300 bp or longer assigned to bacteria in blast+ (bacterial coassemblies). To ensure that we did not lose any bacterial sequences when filtering out eukaryotic contigs, we analyzed taxonomic community structure of all assembly types in Anvi’o (Eren *et al.*, 2020) using the anvi-estimate-scg-taxonomy program and compared the results of full vs. bacterial assemblies. The anvi-estimate-scg-taxonomy program estimates bacterial and archaeal taxonomic composition based on single-copy core genes in the Genome Taxonomy Database (GTDB) (Parks *et al.*, 2020). First, the program determines how many copies of each single-copy gene in the GTDB are present in each assembly. It then uses the most common and abundant single-copy gene across all assemblies to assign taxonomy and calculate coverage for each taxon. In our study, the most common single-copy gene for all assembly types was ribosomal protein S7, so we assigned taxonomy with this gene. To evaluate the sensitivity of taxonomic profiles to single-copy gene choice, we also examined taxonomic profiles generated using the second most common single-copy gene, ribosomal protein S2.

We used the adonis2 function in the vegan package in R (Oksanen *et al.*, 2022) to conduct permutational multivariate analyses of variance (PERMANOVA) to compare the community structure of assemblies which contained only bacterial contigs to those using all contigs. Our response variables were Bray-Curtis dissimilarity matrices generated using vegan, and our predictor variable was contig type. We ran models with 9,999 permutations and found no significant differences in taxonomic community structure between assemblies containing all or only bacterial contigs (individual assemblies: *F*_1,61_ = 0.03, *p* = 0.99; coassemblies: *F*_1,18_ = 0.003, *p* = 0.99; Fig. S2); in fact, for ribosomal protein S7, community structure was identical between contig types (Fig. S2C and D). Consequently, for our results and discussion we focus on taxonomic community structure analyses conducted using assemblies containing bacterial contigs only. Moreover, we likewise used assemblies containing only bacterial contigs to analyze queen gut microbiota metabolism.

We analyzed metabolic potential at the contigs level using Anvi’o’s anvi-estimate-metabolism program (Veseli *et al.*, 2023) which uses the KO database from the Kyoto Encyclopedia of Genes and Genomes (KEGG) (Kanehisa & Goto, 2000; Kanehisa *et al.*, 2012). We included all modules with a pathwise module completeness of >0.5 in our analysis, removed modules assigned to the “Signature modules” class and “Carbon fixation” subcategory, and calculated the relative coverage for each remaining module using average coverage values provided in the anvi-estimate-metabolism output. We then used the program anvi-compute-metabolic-enrichment with our individual assemblies to identify KEGG modules (i.e., metabolic pathways) that were consistently present (i.e., enriched) in some treatments and primarily absent in others (Shaiber *et al.*, 2020). For this function, we set the module completion threshold to 0.5.

### qPCR for 16S rRNA Gene Copy Number

We used qPCR to obtain 16S rRNA gene copy numbers from all gut samples. We generated a four-step standard curve of known 16S rRNA gene copy numbers using DNA extracted from NEB^®^ 5-alpha competent *Escherichia coli* K-12 which we ran in triplicate on every plate. We diluted gut DNA extracts 1:150 in nuclease-free water and ran all samples in triplicate following the protocol in Motta et al. (2018). We used BioRad^®^ SsoFast EvaGreen Supermix with a BioRad^®^ CFX96 real-time system and C1000 thermocycler, and analyzed run data with Bio-Rad^®^ CFX Maestro software (v.2.3).

### Statistical Analyses

We conducted all statistical analyses in R v4.3.1 (R Core Team, 2023). We fit all linear models described in this section using the “lm” function in the base R stats package and examined all model assumptions using the performance package (Lüdecke *et al.*, 2021). We performed any post-hoc tests of linear models using the “TukeyHSD” function in the stats package. When we included natal colony in models, we coded it as a fixed factor as it did not have enough levels to be coded as random. We visualized all data using the ggplot2 package (Wickham, 2016).

We used a series of linear models to compare queen weight and weight change across treatments during different experimental periods. First, we compared weight before diapause and weight change from eclosion to diapause entry between all treatments to ensure that treatments contained queens of similar physical condition. Second, we compared weight at the end of diapause and weight change during diapause between the four-month diapause, recovery control, and recovery + glyphosate treatments to test whether queens in these treatments finished diapause in similar condition. Lastly, we compared weight change after diapause (i.e., during the recovery period) and weight at the end of recovery between the two recovery treatments to determine whether these measurements were affected by glyphosate exposure. Each model contained weight or weight change during the relevant time period as the dependent variable and treatment and natal colony ID as independent variables. We analyzed sugar solution consumption during the recovery experiment using a linear model with volume consumed in the past 24 hours as the dependent variable and natal colony and an interaction between day (coded as categorical) and treatment as independent variables; the interaction was insignificant and was dropped from the final model.

We analyzed how 16S rRNA gene copy counts and the number of paired reads post-filtering varied across treatments using linear models with log_10_ 16S rRNA gene copy count and log_10_ number of paired reads as the dependent variables, respectively, and treatment and natal colony ID as independent variables. For the 16S rRNA gene copy model, we also ran a model without an outlier in the recovery control treatment. We also tested whether 16S gene copy counts were correlated with the number of reads post-filtering using Pearson’s product-moment correlation.

We calculated alpha diversity, specifically the Shannon index, for bumblebee queen gut microbiotas using the “diversity” function in the vegan package (Oksanen *et al.*, 2022). We then used a linear model to determine whether alpha diversity varied with treatment, with Shannon index as the dependent variable, and treatment and natal colony ID as independent variables. As data for this model appeared heteroscedastic, we also ran this model with a ranked dependent variable.

We used the “adonis2” function in the vegan package to conduct PERMANOVAs to analyze how queen gut microbial community structure varied with treatment (Oksanen *et al.*, 2022). We ran models with 9,999 permutations. Our response variables were Bray-Curtis dissimilarity matrices generated using vegan, and our independent variables were treatment and natal colony ID. We evaluated homogeneity of group dispersions using the “betadisper” function in the vegan package with type set to “median” (Oksanen *et al.*, 2022). We conducted post-hoc comparisons using the pairwiseAdonis package for PERMANOVA models (Martinez Arbizu, 2020) and the “TukeyHSD” function for group dispersions. To identify which microbial taxa were differentially abundant across treatments, we conducted an analysis of community structures of microbiomes with bias correction (ANCOMBC). For this, we used the “ancombc2” function in the ANCOMBC package (Lin & Peddada, 2020) with Bonferroni correction and an alpha of 0.05. Before running the analysis, we added a small constant (+1) to all zero values in the queen ID x taxon matrix to obtain p-values and test statistics for taxa which contained structural zeros. We included treatment and natal colony ID as fixed effects for the model.

We investigated how treatment affected KEGG module category composition (i.e., high-level metabolic composition) in queen gut microbiotas using the “adonis2” function in the vegan package (Oksanen *et al.*, 2022). We ran the PERMANOVA with 9,999 permutations. Our response variable was a Bray-Curtis dissimilarity matrix of module category coverages generated using vegan, and our independent variables were treatment and natal colony ID. We evaluated homogeneity of group dispersions using the “betadisper” function in the vegan package with type set to “median” (Oksanen *et al.*, 2022). To examine which specific KEGG modules (i.e., metabolic pathways) were differentially enriched across treatments, we used the Anvi’o program anvi-compute-metabolic-enrichment with the module completion threshold set to 0.5 (Eren *et al.*, 2020; Shaiber *et al.*, 2020). To be conservative, we used unadjusted p-values to determine significance as adjusted q-values were approximately an order of magnitude less than unadjusted p-values.

## Results

### Queen Weight and Sugar Solution Consumption

We found no significant differences in pre-diapause weight or relative weight change between treatments (all *F*_4,18_ < 0.6, all *p* > 0.65; Fig. S3A,D), nor in weight after diapause or weight loss during diapause between four-month and recovery treatments (all *F*_2,10_ < 0.9, all *p* > 0.40; Fig. S3B,E), indicating that queens in all treatments began and ended diapause in similar physical condition.

We also found no difference in weight change during recovery or weight after recovery between recovery treatments (all *F*_1,6_ < 0.03, all *p* > 0.80; Fig. S3C,F). Queens from natal colony 1 weighed more than queens from other natal colonies before diapause (natal colony ID: *F*_2,18_ = 5.13, *p* = 0.02), but there were no differences between queens from different natal colonies for any other metrics (all *F* < 1.7, all *p* > 0.2). Daily sugar solution consumption by queens during the recovery experiment did not differ by treatment (*F*_1,60_ = 0.84, *p* = 0.36), but did differ by day (*F*_6,60_ = 3.07, *p* = 0.011; Fig. S4A) and natal colony ID (*F*_2,60_ = 18.72, *p* < 0.001; Fig. S4B); sugar solution consumption was significantly higher on Day 6 than Day 2 (Tukey HSD, *p* = 0.004) and was significantly lower in queens from natal colony 2 overall (all *p* < 0.001).

### Sequencing and Metagenomic Assemblies

Individual metagenomic assemblies had a mean length of 3.2 million base pairs (bp), contained ~9,200 contigs on average, and had a mean N50 and L50 of 15,500 bp and 180 contigs. However, average quality metrics for assemblies varied with treatment both for individual assemblies (Table S2) and coassemblies by treatment (Table S3), with pre-diapause assemblies having the best quality statistics on average and four-month diapause assemblies having the worst.

After selecting bacterial contigs only, the number of contigs in individual assemblies decreased by 88% on average (range: 46.7 – 99.9%), but the size of the longest contig in each assembly only decreased by an average of 27% (range: 0 – 81.4%), and N50 values for all assemblies increased (Tables S4). For coassemblies, the number of contigs decreased by 87% on average (range: 73.8 – 97.5%) after selecting for bacterial contigs, but the size of the longest contig in each assembly was constant and all N50 values increased (Table S5).
